# The diagnostic potential of combined quantitative polymerase chain reaction and next-generation sequencing using the same primers for periprosthetic joint infection

**DOI:** 10.1128/spectrum.04128-25

**Published:** 2026-07-28

**Authors:** Narumi Ueda, Jun Inoue, Kazuyuki Okuda, Hirokazu Iida

**Affiliations:** 1 Department of Orthopedic Surgery, Japanese Red Cross Wakayama Medical Center, Komatsubara-dori, Wakayama City, Wakayama, Japan; 2 Division of Gastroenterology, Department of Internal Medicine, Kobe University Graduate School of Medicine, Chuo-ku, Kusunoki-cho 38303 , Kobe, Hyogo, Japan; 3 Department of Genome Analysis Center, Kansai Medical University Medical Center, Kansai Medical University, Fumizono‐cho 50196, Moriguchi, Osaka, Japan; 4 Department of Rehabilitation, Kansai Medical University, Uyamahigashi-cho 12880, Hirakata, Osaka, Japan; Meijo University, Nagoya, Japan; IRCCS Galeazzi Orthopedic Institute, Milan, Italy; Meijo Daigaku Yakugakubu Daigakuin Yakugaku Kenkyuka, Nagoya, Japan

**Keywords:** quantitative polymerase chain reaction, next-generation sequencing, periprosthetic joint infection, molecular diagnostics, oral bacteria

## Abstract

Next-generation sequencing (NGS) enables the detection of specific pathogens unidentifiable by conventional cultures, but its application in orthopedics remains inconsistent due to background contamination and irreproducible findings. This study evaluated the diagnostic performance of a novel workflow combining broad-range 16S rRNA gene quantitative PCR (qPCR) screening with downstream NGS, focusing on bacterial biomass thresholds. The qPCR assay demonstrated excellent intrarater reliability, with an intraclass correlation coefficient (ICC) of 0.961 (95% confidence interval, 0.881 to 0.997). Based on serially diluted positive controls, a quantitative threshold of 10⁵ CFU/mL was established as the minimum concentration required for the consistent detection of fastidious taxa, such as *Escherichia coli*. When evaluated against conventional cultures using 95 sonicate fluid and 276 pre/intraoperative tissue samples, the qPCR assay achieved a sensitivity of 80% and a specificity of 72%. Subsequent NGS sequencing of 26 clinical samples and 9 controls showed concordance in 4 of 6 culture-positive infected cases with NGS taxonomy, whereas the remaining discrepancies were likely attributable to culture-based phenotypic misidentification. Notably, among the qPCR-positive cases, three were culture-negative, including two hip prosthesis loosening cases exhibiting polymicrobial profiles, and one post-traumatic osteoarthritis case harboring low-level *Staphylococcus*. Crucially, this post-traumatic patient developed delayed periprosthetic joint infection (PJI) 2 years post-surgery, with cultures identifying *Staphylococcus* previously detected by the initial NGS analysis. Integrating qPCR screening with targeted NGS effectively refines pathogen identification, filters environmental artifacts, and overcomes the diagnostic limitations of culture-negative infections in orthopedic practice.

**IMPORTANCE:**

Next-generation sequencing (NGS) enables the detection of specific pathogens in clinical samples that are not identifiable by conventional methods. However, NGS applications in orthopedics have not been quantitatively evaluated, and findings have been inconsistent owing to contaminants and the presence of non-credible causative organisms. These factors primarily stem from the failure to evaluate low-biomass samples and the absence of proper controls, such as negative controls or mock community DNA samples. This study demonstrates that interpreting results from low-biomass samples requires careful consideration because NGS relies on relative bacterial abundances; distinguishing likely pathogens from contaminants is particularly challenging when bacterial loads are low. We demonstrated that combining NGS with quantitative PCR (qPCR) and applying a Cq cutoff can reduce false positives.

## INTRODUCTION

Routine diagnostic testing may miss a significant number of patients with asymptomatic infections, and these cases are often misdiagnosed as aseptic loosening (1, 2). In clinical practice, when an artificial joint loosens without the typical symptoms of a periprosthetic joint infection (PJI) and a definitive diagnosis cannot be achieved through conventional culture tests, molecular biological techniques, such as next-generation sequencing (NGS), are used to diagnose the infection (3–17). Historically, various testing methods have been explored to detect subclinical pathogens that cannot be identified through traditional culture tests (9–13). To improve the sensitivity of culture testing using sonication and address culture-negative PJIs (12, 13, 18), contemporary molecular frameworks have increasingly incorporated multiple genetic assays (12–14, 17). Although the 2018 Musculoskeletal Infection Society (MSIS) guidelines (19) mention NGS, they lack detailed, quantitative protocols, and even when NGS is utilized, it can be challenging to determine whether the results indicate a true infection. A fundamental challenge in relying on qualitative NGS methodologies, particularly when applied to clinical samples with low microbial biomass, is that they may contain bacteria below the detection limit, or no bacteria at all. Particularly, false positives have been frequently reported with NGS when bacterial loads are low in orthopedic NGS articles (4–7). Consequently, NGS methodologies in orthopedics require more quantitative evaluation to overcome inconsistent results caused by potential contaminants and the presence of non-pathogenic or non-credible causative organisms. Because reagent and laboratory contamination can critically affect sequence-based microbiome analyses (9–11), previous reports (4–7) on the use of NGS methods to diagnose PJI may have been affected by contamination-related inconsistencies. This technical limitation highlights an important methodological gap in current molecular diagnostics: the absence of quantitative assessments and documentation, and a failure to incorporate positive benchmarks. To address these critical diagnostic limitations in low-biomass environments, the primary objective of this study was to establish and validate a pragmatic, highly reproducible molecular diagnostic workflow for PJI by utilizing identical universal primers for both broad-range 16S rRNA gene quantitative PCR (qPCR) screening and downstream NGS. Specifically, we aimed to (i) determine a statistically optimized, parameter-driven qPCR Cq threshold capable of identifying the precise tipping point where biological signals become dominated by environmental contamination; (ii) evaluate the diagnostic performance of this integrated quantitative pipeline against conventional microbiological cultures in a clinical cohort; and (iii) demonstrate the clinical utility of this molecular framework in uncovering occult subclinical pathogens and preventing the underdiagnosis or misclassification of low-biomass implant infections.

## MATERIALS AND METHODS

### Study design and setting

#### Laboratory evaluation of qPCR

**Fig 1:**
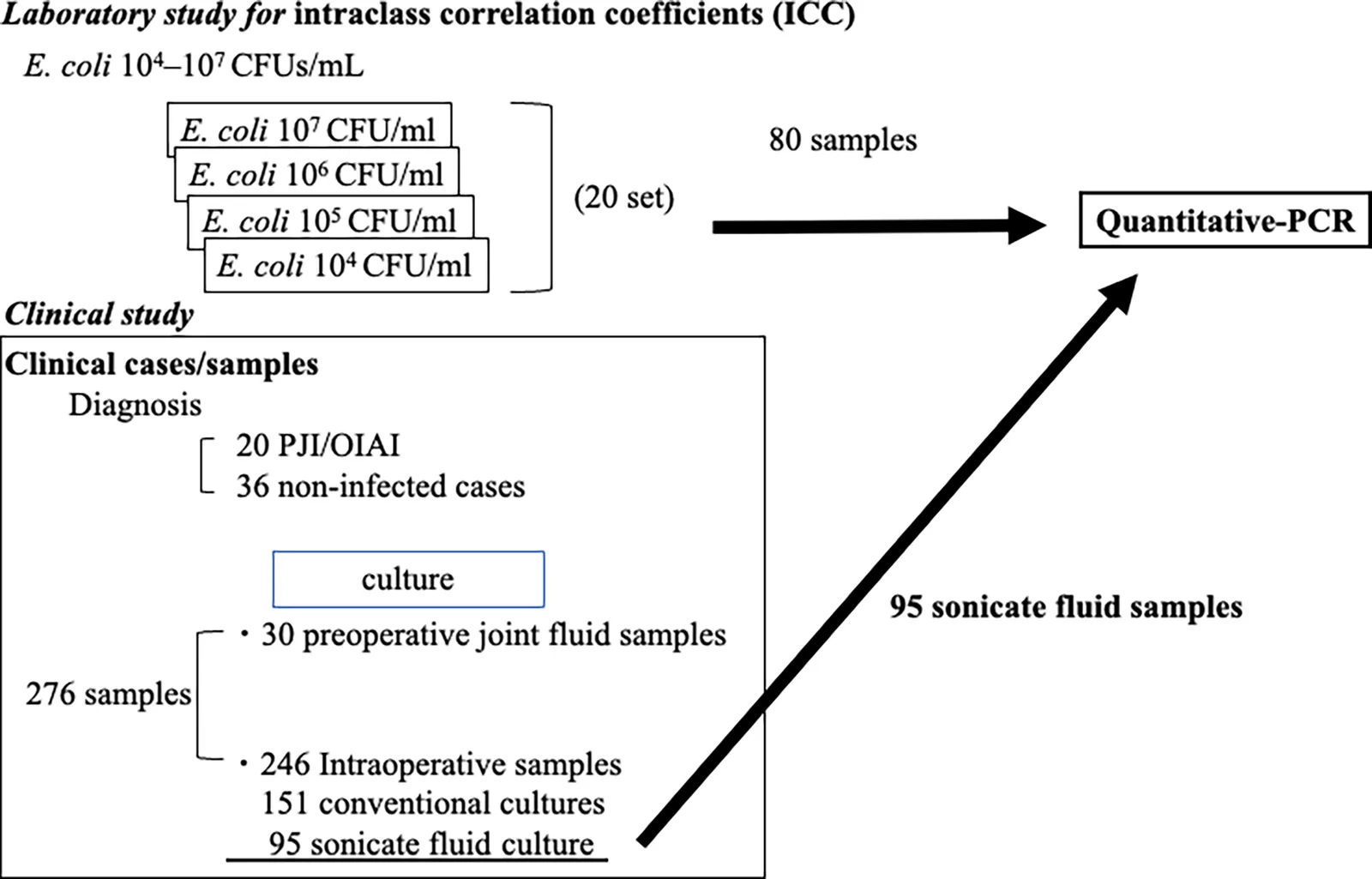
Research method; PCR.

To evaluate the reliability of our qPCR protocol, we used *Escherichia coli* (ATCC 25922) as a quantitative positive control. In a preliminary validation, *E. coli* colonies were cultured in 0.9% sterile saline until the turbidity reached the 0.5 McFarland standard. This strain was selected due to its established reproducibility in quality control standardizations (12–14). Bacterial suspensions were adjusted to final concentrations ranging from 10⁴ to 10⁷ CFU/mL. A total of 80 samples (20 replicates for each of the four concentrations) were prepared to validate the qPCR protocol (see Fig. 1). We assessed the consistency of the quantification cycle (Cq) values and the overall reproducibility of the assays using these samples.

#### Clinical evaluation of qPCR

A total of 95 sonicated fluid samples were obtained from 56 consecutive patients (21 males and 35 females) who underwent implant removal or debridement at our institution between May 2015 and March 2017. These patients were enrolled in a prospective cohort study (see Table 1). Inclusion criteria targeted cases requiring implant removal for which culture testing was performed, and for which the residual sonication (18) fluid was properly preserved in the clinical laboratory. Samples for qPCR were taken from this residual fluid and stored in the microbiology laboratory. First, we determined the optimal Cq thresholds for identifying orthopedic implant-associated infection (OIAI) using these samples. Then, we evaluated the diagnostic performance of qPCR compared with conventional culture results.

**TABLE 1:** Clinical characteristics of the 56 patients enrolled in the qPCR study^*a*^

Characteristic	Result		
	OIAI	Non-infection	*P* value^*c*^
No. of patients (no. of cases)	20 (20)	36 (36)	
No. of sonicate fluid samples (*n* = 95)	37	58	
Median patient age, yrs (IQR)	67 (57–80)	67 (57–77)	0.632^*d*^
Gender, male/female	11/9	10/26	0.044^*f*^
Median follow-up, months (IQR)	37 (33–40)	41 (31–48)	0.051^*d*^
The median time from last operation, yrs (IQR)	4.7 (1.2–10.3)	11.5 (2.9–19)	0.163^*d*^
No. (%) of complications of infection/re-infection until final follow-up	0	1^*b*^	
Joint (%)			
Hip	15 (75)	32 (89)	0.164^*e*^
Knee	2 (10)	2 (5.5)	0.451^*e*^
Others (elbow 1, ankle 2, spine 1, shoulder 1)	3 (15)	2 (5.5)	0.238^*e*^
Type of surgery			
Debridement with implant retention or removal	13	0	0.000^*e*^
Removed implant without debridement	0	3	0.258^*e*^
One-stage exchange	7	27	0.003^*f*^
Primary total hip arthroplasty	0	6	0.06^*e*^
The previous surgery			
Salvage arthroplasty	2	4	0.593^*e*^
Primary arthroplasty (hip, knee, elbow)	10	20	0.690^c^
Bipolar hemi-arthroplasty	2	2	0.428^*e*^
Osteotomy/implant fixation (included spinal instrumentation)	6	10	0.86^*f*^
Sonicate-fluid sample (*n* = 95)			
Artificial joint materials	23	40	0.494^*f*^
Osteosynthesis device	11	16	0.821^*f*^
Cement	2	0	0.149^*e*^
Bone/soft tissue	1	2	0.665^*e*^
Presence of sinus tract, no. of cases	3	0	0.041^*e*^
Peri-implant purulence	11	0	<0.001^*e*^
Antibiotic recipients before sampling, *n* (%)	10 (50)	0	<0.001^*e*^

^*a*^ OIAI, orthopedic implant-associated infection; PJI, periprosthetic joint infection; IQR, interquartile range; THA, total hip arthroplasty.

^*b*^ One patient in the non-infection group experienced a complication categorized as infection/reinfection during the final follow-up period.

^*c*^ *P* values were calculated to compare the OIAI/PJI group and the non-infection group.

^*d*^ Mann-Whitney *U* test.

^*e*^ Fisher's exact test.

^*f*^ Pearson’s chi-squared test.

#### Clinical evaluation of NGS

**Fig 2:**
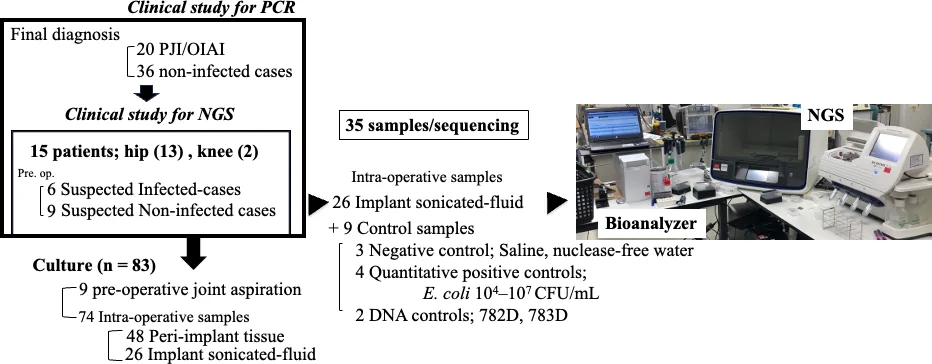
Research method; next-generation sequencing.

Of the samples used for qPCR evaluation, 26 sonicate fluid samples were obtained from 15 consecutive patients (6 males, 9 females) who underwent implant removal or debridement at our institution between May 2015 and October 2015. These patients were enrolled in this prospective cohort NGS study (see Fig. 2). The cohort comprised 13 hip and 2 knee cases, which included 4 infected cases (3 PJIs and 1 fracture-related infection (FRI) (20)) and 11 non-infected cases (see Table 2). All included cases required implant removal, underwent sonicated fluid culture (SFC) testing, and had residual sonication fluid (18) properly preserved in the clinical laboratory.

**TABLE 2:** Clinical characteristics of the 15 patients enrolled in the NGS study^*a*^

Case no.	Age	Sex	CRP (mg/L)	Observation (months)	Reason for surgery	Joint	Received antibiotics	Aspiration joint fluid (preoperative) culture
1	64	F	276	95	PJI	Hip	+	*Streptococcus* sp.
2	73	F	3	53	Loosening	Hip	−	–
3	55	M	92	40	FRI	Hip	−	CNS
4	83	M	11	72	PJI	Hip	−	–
5	58	F	1	99	Loosening	Hip	−	ND
6	70	M	30	99	PJI	Knee	+	CNS^＊^
7	50	M	5	76	OA	Hip	−	–
8	48	M	2	102	Dislocation	Hip	−	ND
9	46	M	1	99	OA	Hip	−	–
10	82	F	3	15	OA	Hip	−	–
11	61	F	0	101	Loosening	Hip	−	–
12	73	F	1	101	Loosening	Knee	−	ND
13	69	F	1	106	OA	Hip	−	ND
14	82	F	1	36	OA	Hip	−	ND
15	74	F	2	93	Pain	Hip	−	ND

^*a*^ NGS, next-generation sequencing; PJI, prosthetic joint infection; FRI, fracture-related infection; OA, osteoarthritis; CRP, C-reactive protein; ND, not done; +, presence; −, absence. *Coagulase-negative *Staphylococcus* sp.

Samples for qPCR and NGS were aliquoted from the remnant sonication fluid and stored in the microbiology laboratory. In total, 26 clinical samples and nine controls were analyzed in a single sequencing run. The controls consisted of four quantitative positive controls (*E. coli*, 10⁴–10⁷ CFU/mL), three negative controls (one nuclease-free [NF] water sample for library preparation and two sterile saline samples for sonication), and two mock community DNA (mock) samples (12–14, 21–25) (Fig. 2). The relationship between the qPCR and NGS results was subsequently compared with the culture test results.

The primary objective was to analyze clinical specimens with varying bacterial DNA levels to identify which bacteria predominate under low-biomass conditions and which are likely contaminants. The secondary objective was to determine the lowest concentration at which bacteria could be consistently detected as the dominant organism, representing the highest proportion of NGS reads. Based on our previously proposed quantitative framework (14), we utilized the corresponding Cq value as a threshold to define reliable detection, whereby the targeted bacterium is identified as the dominant taxon in the NGS results. Using this definition, we validated this cutoff point and assessed the ability of NGS to distinguish true biological signals from background contamination.

Third, clinical samples were divided into low-biomass and high-biomass groups according to this cutoff value. For each group, we analyzed the top ten bacterial taxa identified by NGS and evaluated their correlation with quantitative measurements. Furthermore, we reassessed the top ten bacterial genera after applying bioinformatic decontamination tools (15, 16).

Finally, patients underwent long-term clinical follow-up to monitor their clinical courses, particularly for cases where definitive conclusions could not be drawn solely from the acute clinical presentation and NGS results.

### Pre/postoperative assessment

Preoperatively, synovial aspirates were obtained from patients exhibiting clinical evidence of infection, such as elevated serum C-reactive protein levels, persistent or increasing pain, implant loosening, and fever. OIAI, which includes PJI and FRI, was diagnosed according to the MSIS criteria (19) and the FRI descriptive flowchart (20).

### Intraoperative sample collection

During surgery, multiple peri-implant tissue sites were sampled for histological analysis, and implants were removed under sterile conditions. Individual components of the extracted implants and adjacent bone or tissue were separately subjected to sonication (18). Other peri-implant specimens were transferred to the institutional microbiology laboratory for routine culture (18). Additionally, peri-implant tissues were evaluated intraoperatively using frozen section analysis (18, 19).

### Antibiotic treatment

Routine antibiotic prophylaxis comprised 1 g of cefazolin administered intravenously at the induction of anesthesia and at 3-h intervals throughout surgery (18). If a pathogen had been detected prior to surgery, targeted antibiotic treatment was initiated after intraoperative tissue sampling, guided by antimicrobial susceptibility testing results. Otherwise, empirical antibiotic treatment was administered post-sampling. Previous antimicrobial therapy was defined as the administration of antimicrobial agents for at least 1 day within the 14 days preceding implant removal (18). In cases of suspected infection, preoperative antibiotics were withheld until culture samples had been collected.

### 16S rRNA gene qPCR

To facilitate post-sequencing analyses, qPCR was performed using serially diluted, known quantities of spiked input DNA (10, 12–14). The qPCR threshold used for clinical screening differed from the more stringent threshold adopted for downstream NGS interpretability analyses. The V1–V2 region of the 16S rRNA gene was selected to ensure accurate identification of a broad range of clinically relevant bacteria (12, 21, 22, 24, 25). Broad-range universal primers targeting this region were 8F (5′-AGAGTTTGATCCTGGCTCAG-3′) and 338R (5′-TGCTGCCTCCCGTAGGAGT-3′) (12–14, 24). Bacterial DNA was extracted from sonicate fluid samples using the QIAamp DNA Mini Kit (QIAGEN, Hilden, Germany) according to the manufacturer’s protocol. Broad-spectrum qPCR assays were conducted with SYBR Green on a Light Cycler Nano instrument (Roche Diagnostics, Basel, Switzerland) using the FastStart Essential DNA Green Master (Roche Diagnostics, Burgess Hill, UK). The thermocycling conditions comprised an initial denaturation at 95°C for 10 min; 40 cycles of denaturation at 94°C for 1 min, annealing at 58°C for 1 min, and extension at 68°C for 1 min; and a final extension at 95°C for 10 s. Cq values were calculated using the LightCycler Nano Software (Roche Molecular Systems, Inc., Pleasanton, CA, USA).

### Next-generation sequencing

Initially, DNA was amplified via PCR using custom primers targeting the V1–V2 region (12–14, 24), identical to those used for 16S rRNA gene qPCR. To achieve optimal amplicon yields and minimize background artifacts based on negative-control qPCR findings, the thermocycling profile was reduced from 40 to 30 cycles. Amplicon libraries were prepared using the Ion Plus Fragment Library Kit (Thermo Fisher Scientific, Waltham, MA, USA), barcoded with IonXpress Barcode Adapters (Thermo Fisher Scientific), and processed according to the Ion Amplicon Library Preparation (Fusion Method) protocol (26).

Purification was performed using Agencourt AMPure XP beads (Beckman Coulter, High Wycombe, UK), and DNA concentration was quantified via capillary electrophoresis using an Agilent 2100 Bioanalyzer (Agilent Technologies, Inc., Santa Clara, CA, USA). Sample DNA was subsequently loaded onto Ion Sphere Particles for emulsion PCR using the Ion Chef instrument, the Ion PGM Hi-Q View Chef 400 Kit, and the Ion 318 Chip v2 (Life Technologies, Carlsbad, CA, USA). Sequencing was performed on the Ion Torrent PGM platform (Thermo Fisher Scientific) (4–7, 12–14, 21, 22, 25, 26).

### Bioinformatic processing of 16S rRNA gene sequencing data

Sequence data were quality-filtered and analyzed using QIIME (27) version 1.8.0. For comparative analysis, a total of 10,000 reads were randomly subsampled per specimen. Chimeric sequences were identified and removed using USEARCH version 6.1.544, and operational taxonomic units (OTUs) were clustered at a 97% sequence similarity threshold using UCLUST. Taxonomic assignment was performed against the Greengenes reference database (version 13.8). To minimize non-bacterial off-target signals, a post-classification organellar filtering step was applied. OTUs assigned to chloroplast or mitochondrial lineages—defined a priori by taxonomy strings containing “*c__Chloroplast*” or “*Mitochondria*”—were identified from the taxonomy OTU table and removed from the BIOM table prior to secondary analyses. This filtering was applied uniformly to all organellar OTUs and was not restricted to *Streptophyta*-labeled reads. Following organellar filtering, a prevalence-based contamination analysis was conducted using the R package *decontam* (15–17) as an exploratory yet reproducible computational assessment of candidate contaminant OTUs. The three negative controls (two sterile saline controls and one NF water control) were defined as negative references for the algorithm. Prevalence thresholds of 0.1, 0.2, 0.3, 0.4, and 0.5 were systematically evaluated. Because the top mock-community OTUs were retained at a threshold of 0.3 but began to be classified as candidate contaminants at thresholds of 0.4 and above, 0.3 was selected as the strictest threshold to preserve the expected mock-community signal (see Fig. S1). OTUs identified as candidate contaminants at this selected threshold were subsequently removed before secondary comparisons.

### Mock bacterial community

To evaluate sequencing accuracy, mock community genomic DNA was amplified and sequenced in parallel with the clinical samples. The following reagents were obtained from BEI Resources: Genomic DNA from Microbial Mock Community B (Even, Low Concentration 1), v5.1L (HM-782D) and Genomic DNA from Microbial Mock Community B (Staggered, Low Concentration 1), v5.1L (HM-783D). These mock samples contain 17 genera (20 species) provided at even and staggered concentrations, respectively (12–14, 21–25).

### Quantitative positive control

Controls generated by serial dilution of DNA of known composition were included to determine the biomass level at which contamination becomes a dominant feature of sequencing results (10, 12–14). NGS microflora data from mock samples previously demonstrated a low relative abundance of Gram-negative bacteria when primers targeting the V1–V2 variable regions of the 16S rRNA gene were utilized (14, 25). Therefore, we selected *E. coli* (ATCC 25922) as a model organism to evaluate its sequencing-based detectability (12–14). Simulated infection models with varying bacterial concentrations were prepared using *E. coli* suspended in sterile saline. Briefly, *E. coli* was cultured in broth medium overnight, diluted with sterile saline, and adjusted based on McFarland standards to yield four distinct concentrations: 10⁴, 10^5^, 10^6^, and 10^7^ CFU/mL (12–14).

### Negative control

Negative controls comprised three samples: two aliquots of sterile saline (serving as background controls for the sonication process and the dilution of the quantitative positive control) and one aliquot of NF water processed identically through the NGS workflow (12–14). These three samples were utilized not only for the descriptive characterization of background taxa but also as designated negative references for the prevalence-based *decontam* analysis (15, 16).

### Strategies for the identification of pathogenic and contaminant bacteria

For culture-positive specimens obtained from the same patient, a bacterium was classified as the causative pathogen if the bacterial count was ≥10⁵ CFU/mL and the organism was detected in the post-decontamination NGS results. Similarly, if the same bacterium was quantitatively detected across two or more NGS samples, it was defined as the causative pathogen. In cases where only a single sample was qPCR-positive, the bacterium was deemed the causative pathogen if it accounted for 100% of the total sequencing reads. Conversely, it was classified as a contaminant if its relative abundance was equal to or less than the baseline percentage determined prior to running the *decontam* algorithm (15, 16). All remaining bacterial taxa were categorized as contaminants.

### Statistical analysis

Correlations were evaluated using Spearman’s rank correlation coefficient. Test reliability was assessed using generalizability theory based on intraclass correlation coefficients (ICCs), which were interpreted as follows: <0.40, poor; 0.41 to 0.75, fair to good; and >0.75, excellent (28). For the regression equation, the coefficient of determination was obtained via analysis of variance, and prediction accuracy was evaluated. The clinical sample size for qPCR was determined based on previous reports (6, 18). Assuming a 30% difference in sensitivity between molecular methods and culture, a power of 80%, and a two-sided alpha error of 0.05, a minimum sample size of 55 patients was required. The Shapiro–Wilk test was used to assess data normality. Continuous variables were compared between groups using Student’s *t*-test or the Mann–Whitney U test, as appropriate. Categorical variables were evaluated using 2 × 2 contingency tables and Pearson’s chi-squared test, or Fisher’s exact test if any expected cell frequency was less than 5. All statistical analyses were performed using IBM SPSS Statistics version 31.0 (IBM Corp., Armonk, NY, USA). A *P* value < 0.05 was considered statistically significant for all two-sided tests. A microbial test result was classified as a true positive if it was concordant with any of the infection-defined criteria described above (19, 20). Sensitivity and specificity were calculated for culture testing, qPCR, and their combination, using the infection-defined criteria as the reference standard. The optimal clinical Cq threshold was determined by maximizing Youden’s J statistic, which was calculated as sensitivity + specificity − 1.

Receiver operating characteristic (ROC) analysis was performed using SPSS. The 95% confidence intervals (CIs) for sensitivity and specificity were generated using the Clinical Calculator 1 (18, 29). The sensitivity and specificity of the different diagnostic methods were compared using McNemar’s test.

## RESULTS

### Reproducibility and reliability of qPCR assays

**Fig 3:**
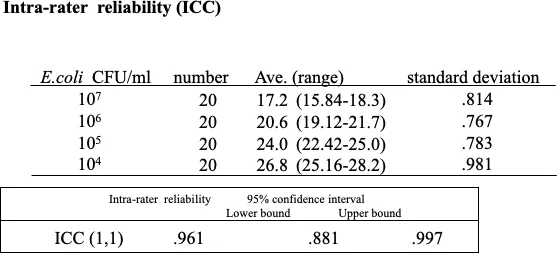
qPCR assay validation. Summary of quantification cycle (Cq) values, standard deviations (SD), and intrarater reliability across serial dilutions of *Escherichia coli* (10^4^–10^7^ CFU/mL; *n* = 20 replicates per concentration). The lower table presents the ICC [1,1] alongside its 95% CIs (lower and upper bounds) to verify measurement consistency.

The qPCR assays demonstrated excellent reproducibility and reliability, with an ICC [1, 1] for Cq values of 0.961 (95% CI, 0.881–0.997) (see Fig. 3). The mean (range) Cq values at *E. coli* concentrations of 10^7^, 10^6^, 10^5^, and 10^4^ CFU/mL were 17.2 (15.84–18.3), 20.6 (19.12–21.7), 24.0 (22.42–25.0), and 26.8 (25.16–28.2), respectively (see Fig. 3).

### Clinical-based Cq cutoff value obtained from statistical analysis

**Fig 4:**
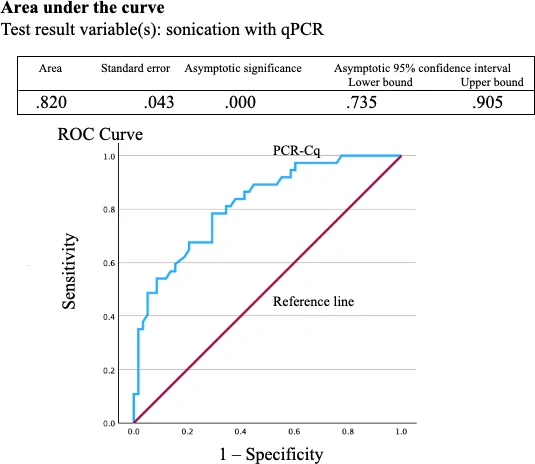
ROC analysis of qPCR Cq values for distinguishing contamination-dominated low-biomass samples from higher-biomass samples. The optimal cutoff (Cq = 26.4) was determined using the Youden index. Area under the curve (AUC), 0.82; 95% CI, 0.74–0.91.

**Fig 5:**
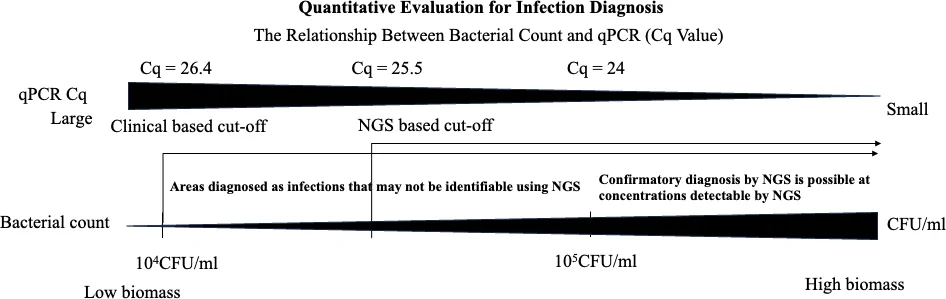
Conceptual diagram illustrating the relationship between bacterial quantity and molecular diagnostic thresholds. The schematic workflow highlights the inverse correlation between qPCR Cq values (top scale) and absolute bacterial counts (bottom scale) across low-biomass and high-biomass conditions. The clinical-based cutoff (Cq = 26.4), corresponding to 10^4^ CFU/mL, defines the threshold for infection diagnosis via qPCR, whereas the NGS-based cutoff (Cq = 25.5), corresponding to 10^5^ CFU/mL, represents the analytical limit for reliable bacterial identification. The diagram delineates two distinct analytical zones: a low-biomass region where specimens diagnosed as infected via qPCR may remain unidentifiable or prone to artifact contamination under NGS single-testing a high-biomass region where NGS can provide confirmatory evidence.

The baseline characteristics and clinical comparisons between the OIAI and non-OIAI groups (see Table 1)—including serum CRP values, histopathological findings, and microbiological culture results—are summarized in Table 3. Data were analyzed from 175 qPCR derivation samples, consisting of 80 laboratory-derived samples and 95 clinical sonicated fluid samples (which included 30 clinical validation samples). The clinical study evaluated a total of 276 culture results obtained from 56 patients (see Fig. 2). These consisted of 30 preoperative joint aspirate cultures and 246 peri-implant tissue cultures; the latter comprised 95 implant SFCs and 151 conventional cultures. Culture results for patients diagnosed with OIAI are summarized in Tables 3 and 4. Sensitivity and specificity were calculated for culture testing, qPCR, and their combination, using the OIAI-defined criteria as the reference standard. Based on the Youden J statistic, the clinical Cq cutoff was determined to be 26.4 with a sensitivity of 0.773 and specificity of 0.765 (see Fig. 4). However, dilution experiments demonstrated that reliable target-organism dominance in NGS profiles was maintained only at bacterial concentrations corresponding to Cq values ≤ 25.5. Therefore, a conservative threshold of Cq ≤ 25.4 was adopted for downstream NGS interpretability analyses (see Fig. 5).

**TABLE 3:** Clinical and microbiological characteristics of the 56 patients enrolled in the qPCR study^*a*^^*,^b^,^d^,*^^*g*^

Characteristic	Result		
	OIAI (*n* = 20)	Non-infection (*n* = 36)	*P* value^*c*^
Sites with tissue evidence of inflammation			
≥5 neutrophils/high-power field (%)	12/14	2/28	<0.001^*f*^
Laboratory findings: serum CRP (mg/L)			
Median (IQR): before operation	21.6 (3.0–85.3)	1.1 (0.45–3.2)	<0.001^*e*^
Sites with CRP > 10.0 mg/ L (%)	14/20 (70)	2/36 (6)	<0.001^*h*^
Preoperative joint aspirate cultures (*n* = 30)			
Sites with > 1 culture positive (%)	10/15 (67)	0/12 (0)	<0.001^*h*^
Detection rate (%)	10/16 (63)	0/13 (0)	<0.001^*h*^
Intraoperative peri-implant tissue cultures (*n* = 151)			
Mean (range) of samples taken	3.5 (1–9)	3 (0–7)	0.601^*e*^
Sites with > 1 culture positive (%)			
All cases	12/20 (60)	1/36 (3)	<0.001^*f*^
Antibiotic recipients before sampling case	5/10 (50)	0	
Detection rate (%)			
All cases	17/79 (22)	0/72 (0)	<0.001^*h*^
Antibiotic recipients before sampling case	10/31 (32)	0	
Sonicate fluid cultures (*n* = 95)			
Median (IQR) of samples taken	2.0 (1.5–2.0)	2.0 (1.0–2.0)	0.107^*e*^
Sites with > 1 culture positive (%)			
All cases	12/20 (60)	0/36 (0)	<0.001^*f*^
Antibiotic recipients before sampling case	5/10 (50)	0	
Detection rate (%)			
All cases	14/37 (36)	0/58 (0)	<0.001^*h*^
Antibiotic recipients before sampling case	7/20 (35)	0	
Sonicate fluid PCR (*n* = 95)			
Sites with > 1 PCR positive (%)			
All cases (Cq ≤ 25.4)	16/20 (80)	10/36 (28)	<0.001^*h*^
Antibiotic recipients before sampling case			
Positive rate (%)	8/10 (80)	0	
All cases (Cq ≤ 25.4)	15/20 (75)	0	

^*a*^ OIAI, orthopedic implant-associated infection; CRP, C-reactive protein; IQR, interquartile range.

^*b*^ Where the denominator is shown, data were not available for all study patients.

^*c*^ *P* values were calculated by comparing definite OIAI groups with non-infection groups.

^*d*^ Patient who received a minimum of 1 day of antibiotic therapy within 14 days prior to removal of the implants.

^*e*^ Mann–Whitney *U* test.

^*f*^ Fisher's exact test.

^*g*^ Student's *t*-test.

^*h*^ Pearson’s chi-squared test.

**TABLE 4:** Diagnostic and microbiological results of individual study subjects with orthopedic implant-associated infections^*a*^

No.	Age	Sex	Joint	Disease/treatment	CRP (mg/L)	Sinus tract	Purulence	Received antibiotics	Histopathology results (*n* = 14)	Culture results^*b*^			PCR results	Term from last operation (yrs)	Follow-up (month)
										Preoperative (*n* = 16)	Peri-implant tissue (*n* = 79)	Sonicate fluid (*n* = 37)	≤25.4^*c*^		
1	70	M	Knee	PJI/2st.	30.0	−	+	+	ND	CNS (1)/1	N (0)/2	CNS (1)/2	P (1)/2	63	37
2	64	F	Hip	PJI/2st.	66.0	−	+	+	+	*Streptococcus* sp. (1)/1	*Streptococcus* sp. (1)/1	*Streptococcus* sp. (2)/2	P (2)/2	1.2	37
3	55	M	Hip	OIAI/debridement	92.0	−	+	−	+	CNS (1)/1	CNS (1)/5	CNS (1)/2	P (1)/2	0.5	40
4	83	M	Hip	OIAI/1st.	11.0	−	−	−	+	N (0)/1	N (0)/3	*Corynebacterium* sp. (2)/2	P (2)/2	3.1	30
5	53	M	Ankle	OIAI/debridement	247.0	+	+	+	ND	ND	MRSA (1)/2	MRSA (1)/3	P (3)/3	0.1	45
6	85	M	Hip	OIAI/debridement	115.0	−	+	+	ND	GBS (1)/1	GBS (1)/2	N (0)/1	P (1)/1	0.2	37
7	72	F	Hip	PJI/1st.	2.3	−	+	−	+	*S. maltophilia* (1)/2	N (0)/3	N (0)/2	N (0)/2	20	51
8	74	F	Hip	PJI/2st.	274.0	−	−	+	+	ND	CNS (2)/2	CNS (2)/2	P (2)/2	1	39
9	20	M	Ankle	OIAI/debridement	0.1	+	+	+	ND	ND	N (0)/2	N (0)/1	N (0)/1	2	33
10	74	F	Hip	PJI/2st.	1.6	−	+	−	+	N (0)/1	N (0)/8	N (0)/3	P (1)/3	9	45
11	76	F	Hip	PJI/1st.	50.5	−	+	+	ND	*Streptococcus* sp. (1)/1	N (0)/5	N (0)/4	P (2)/4	18	38
12	78	F	Hip	PJI/2st.	12.1	+	−	+	+	N (0)/1	*Corynebacterium* sp. (1)/7	N (0)/1	P (1)/1	7	36
13	63	M	HIp	PJI/1st.	154.0	−	+	+	+	*Streptococcus* sp. (1)/1	*Streptococcus* sp. (2)/4	N (0)/3	P (3)/3	9	36
14	43	M	Hip	PJI/2st.	22.3	−	+	−	+	*Campylobacter fetus* subsp. (1)/1	N (0)/6	N (0)/1	N (0)/1	8	34
15	80	F	Hip	PJI/1st.	10.2	−	−	−	+	N (0)/1	*Streptococcus* sp. (3)/9	N (0)/1	P (1)/1	25	24
16	81	F	Knee	OIAI/1st.	20.9	−	−	−	ND	CNS (1)/1	CNS (1)/3	N (0)/1	P (1)/1	1.2	37
17	80	M	Elbow	PJI/1st.	47.5	−	−	+	+	*Streptococcus* sp. (1)/1	N (0)/4	*Streptococcus* sp. (1)/1	N (0)/1	10.7	4
18	87	M	Hip	PJI/1st.	2.5	−	−	−	−	N (0)/1	CNS (2)/5	CNS (2)/2	P (2)/2	1.4	46
19	74	F	Hip	PJI/1st.	4.6	−	−	−	+	ND	CNS (1)/4	CNS (2)/2	P (1)/2	12	35
20	22	M	Hip	OIAI/debridement	1.7	−	−	−	−	ND	MSSA (1)/2	MSSA (1)/1	P (1)/1	1.3	29

^*a*^ PJI, prosthetic joint infection; OIAI, orthopedic implant-associated infection; 1st., one-stage exchange; 2st., two-stage exchange; ORIF, open reduction internal fixation; CRP, C-reactive protein; P, positive; N, negative; ND, not done; +, presence; −, no presence; CNS, coagulase-negative *Staphylococcus* sp.; VGS, viridans group *Streptococcus* sp.; MSSA, methicillin-susceptible *Staphylococcus aureus*; MRSA, methicillin-resistant *Staphylococcus aureus*; *coryne*. sp., Gram-positive bacillus resembling *Corynebacterium* sp.; GBS, Group B *Streptococcus* sp.; *S. maltophilia*, *Stenotrophomonas maltophilia*.

^*b*^ (Number of positive specimens)/Number of samples.

^*c*^ PCR positive criteria; ≤25.4 (Cq value); established analytical cutoff via next-generation sequencing validation.

### Characteristics of sequencing results near the quantitative limit and determination of the NGS cutoff

Mock analysis detected microbes from all 17 target genera. However, the sequencing results demonstrated a low relative abundance of Gram-negative bacteria; specifically, NGS analysis of sample HM-782D identified several Gram-negative taxa with varying relative abundances, including *Acinetobacter* (0.09%), *Enterobacteriaceae* (0.08%), and *Pseudomonadaceae* (0.01%) (see Table S1). Regarding the bacteria identified in both the positive control (serially diluted *E. coli*) and the negative controls, seven of the top ten bacteria in the quantitative positive control (highlighted in bold in Table S2) were also present in the negative controls. In the NGS results for the serially diluted *E. coli*, members of the family *Enterobacteriaceae* were most frequently detected at concentrations ranging from 10^7^ to 10^5^ CFU/mL. However, at 10^4^ CFU/mL, *Enterobacteriaceae* (presumed to represent *E. coli*) was no longer the dominant taxon, accounting for only 4% of the total reads (see Table S2). Consequently, a Cq value of 25.5 (10⁵ CFU/mL) was determined as the operational threshold for reliable bacterial detection. Because higher Cq values reflect lower bacterial biomasses that are prone to contamination, the analytical cutoff value for reliable detection was conservatively established as Cq ≤ 25.4.

### Diagnostic performance of qPCR using the NGS-derived cutoff value

The sensitivities of synovial fluid culture, peri-implant tissue culture, and SFC were 67%, 60%, and 50%, respectively, whereas their specificities were 100%, 97%, and 100%, respectively (see Table 5). In comparison, when applying the NGS-derived cutoff value (Cq ≤ 25.4), the qPCR assay achieved a sensitivity of 80% and a specificity of 72% (see Table 5). To enhance diagnostic accuracy, we evaluated the performance of combining these microbial culture methods with the qPCR assay (see Table 6). Crucially, when defining positivity as either a single positive culture or a positive qPCR result (Cq ≤ 25.4), the combination method achieved the highest sensitivity at 95.0% (95% CI: 73.1%–99.7%), albeit with a reduced specificity of 69.4% (95% CI: 51.7%–83.1%). Conversely, when applying a stricter consensus criterion requiring either two positive cultures or one positive culture plus a positive qPCR result, the combination strategy demonstrated a highly balanced diagnostic profile, achieving a sensitivity of 80.0% (95% CI: 55.7%–93.3%) and a perfect specificity of 100% (95% CI: 88.0%–100%), alongside a positive predictive value of 100% and a negative predictive value of 90.0% (see Table 6).

**TABLE 5:** Comparison of microbiologic test^*a*^^,^^*b*^^,^^*c*^^,^^*d*^^,^^*e*^

Test	Cases (*N* = 56)		Sensitivity	Specificity	Positive predictive value	Negative predictive value
	OIAI	Non-infected				
	No. of patients with positive specimens^b^		(95% CI)			
Preoperative culture						
Synovial fluid	10/15	0/12	66.7 (38.7–87.0)	100 (69.8–100)	100 (65.5–100)	70.1 (44.0–88.6)
Peri-implant tissue culture						
≥1 positive culture	12/20	1/36	60.0 (36.4–80.0)	97.2 (83.8–99.9)	92.3 (62.1–99.6)	81.4 (66.1–91.1)
≥2 positive cultures^*c*^	4/19	0/36	21.1 (7.0–46.1)	100 (90.0–100)	100 (39.6–100)	70.6 (56.0–82.1)
Sonicate fluid culture^*d*^						
≥1 positive culture	10/20	0/36	50.0 (27.9–72.1)	100 (88.0–100)	100 (65.5–100)	78.3 (63.2–88.5)
≥2 positive cultures^*c*^	5/12	0/36	41.7 (16.5–71.4)	100 (88.0–100)	100 (46.3–100)	83.7 (68.7–92.7)
Sonicate fluid PCR^***e***^						
Cq ≤ 25.4						
≥1 positive PCR	16/20	10/36	80.0 (55.7–93.4)	72.2 (54.6–85.2)	61.5 (40.7–79.1)	86.7 (68.4–95.6)
≥2 positive PCR	7/12	1/14	58.3 (28.6–83.5)	92.9 (64.2–99.6)	87.5 (46.7–99.3)	72.2 (46.4–89.3)

^*a*^ OIAI, orthopedic implant-associated infection; PCR, polymerase chain reaction; Cq, quantification cycle. Clinical Calculator 1 was used for the calculation of sensitivity, specificity, positive predictive value, negative predictive value, and their corresponding 95% confidence intervals (18, 29).

^*b*^ Where the denominator is shown, data were not available for all study patients.

^*c*^ The number of cultures positive for the same microorganism is given.

^*d*^ Sonicate fluid culture with (centrifugation ≥ 1 colony-forming unit/plate); the number of colony-forming units per agar plate growing on either aerobic or anaerobic plates (whichever yielded higher counts) is given.

^*e*^ Positive PCR was defined as qPCR with a Cq value of ≤25.4, representing the NGS-derived cutoff.

**TABLE 6:** Comparison of diagnostic performance among combined culture^*a*^^*,*^^*d*^

Test	Cases (*N* = 56)		Sensitivity	Specificity	Positive predictive value	Negative predictive value
	OIAI	Non–infected				
	No. of patients with positive specimens^*b*^		(95% CI)			
Conventional culture method						
(Pre-operative culture and Tissue culture)						
≥1 Positive culture	17/20	1/36	85.0 (61.1-96.0)	97.2 (83.8-99.9)	94.4 (70.6-99.7)	92.1 (77.5-97.9)
≥2 Positive cultures^*c*^	8/20	0/36	40.0 (20.0-63.6)	100 (90.0-100)	100 (59.8-100)	75.0 (60.1-85.9)
Combination culture method						
(Pre-operative culture and tissue culture, sonicate fluid culture)						
≥1 Positive culture	18/20	1/36	90.0 (66.9-98.2)	97.2 (83.8-99.9)	94.7 (71.9-99.7)	94.6 (80.5-99.1)
≥2 Positive cultures^*c*^	14/20	0/36	70.0 (45.7-87.2)	100 (88.0-100)	100 (73.2-100)	85.7 (70.8-94.1)
Combination culture and PCR method (culture method and PCR)						
≥1 Positive culture or PCR positive^*e*^						
cut-off ≤ 25.4 (Cq)	19/20	11/36	95.0 (73.1-99.7)	69.4 (51.7-83.1)	63.3 (43.9-79.5)	96.2 (78.4-99.8)
≥ 2 positive cultures^*c*^						
or one positive culture and 1 PCR positive^*e*^						
Cut-off ≤ 25.4 (Cq)	16/20	0/36	80.0 (55.7-93.3)	100 (88.0-100)	100 (75.9-100)	90.0 (75.4-96.7)

^*a*^ OIAI, orthopedic implant-associated infection; Cq, quantification cycle. Clinical Calculator 1 was used for the calculation of sensitivity, specificity, positive predictive value, negative predictive value, and their corresponding 95% confidence intervals (18, 29).

^*b*^ Where the denominator is shown, data were not available for all study patients.

^*c*^ The number of cultures positive for the same microorganism is given.

^*d*^ Sonicate fluid culture with centrifugation (≥1 colony-forming unit (CFU)/plate); the number of CFUs per agar plate growing on either aerobic or anaerobic plates (whichever yielded higher counts) is given.

^*e*^ Positive PCR was defined as qPCR with a Cq value of ≤25.4, representing the NGS-derived cutoff.

### Clinical characteristics and diagnostic outcomes of the NGS cohort

A subset of patients from the qPCR-tested group who underwent implant removal within a defined timeframe was selected for subsequent NGS analysis. This cohort included 83 culture results from 15 patients: 6 with suspected infection and 9 with suspected non-infected loosening based on preoperative clinical records. Patient-level diagnostic findings, organism-level concordance between culture and NGS, and the diagnostic performance of sonicate fluid qPCR and qPCR combined with the NGS criterion in the 15-patient NGS cohort are summarized in Tables 7–9. These results comprised 9 preoperative joint aspirate cultures, 26 SFCs, and 48 conventional peri-implant tissue cultures (see Fig. 2). Three of the nine preoperative joint aspirate cultures were positive, all of which were confirmed as true infections according to postoperative diagnostic criteria (see Table 2). Among the 15 patients, 5 had positive cultures from either preoperative aspirates or surgical specimens; 4 of these 5 patients were ultimately diagnosed with infection based on clinical criteria (see Table 7).

**TABLE 7:** Detailed intraoperative microbiological, histopathological, and molecular results for the 15 patients in the NGS cohort^*a*^

Case	Suspected infection: Yes/No (preoperative clinical records)	Diagnosis with 2018 MSIS/ICM		Implications of molecular testing	Equivocal results with culture only	Histopathology	Peri-implant culture^*b*^	Sonicated culture results		Cq (qPCR)
		Culture-based diagnosis	Combination with qPCR and NGS					Sonicated sample type	Sonicate fluid culture^*b*^	
1	Yes	Infection: PJI	Infection: PJI	Confirmed	No	Positive	(0)/1	Artificial joint (2)	*Streptococcus* (2)/2	17.7, 18.7
2	Yes	Non.	Non.	Suggestive	Yes	Negative	(0)/7	Artificial joint (2)	(0)/2	21.0, 27.4
3	Yes	Infection: FRI	Infection: FRI	Confirmed	No	Positive	CNS (1)/5	Nail, peri-implant tissue	CNS (1)/2	28.5, 21.4
4	Yes	Infection: PJI	Infection: PJI	Confirmed	No	Positive	(0)/3	Artificial joint (2)	*Corynebacterium* (2)/2	21.6, 25.6
5	Yes	Non.	Non.	Suggestive	Yes	Negative	(0)/4	Artificial joint (2)	(0)/2	23.4, 30.8
6	Yes	Non.	Infection: PJI	Confirmed	Yes	ND	(0)/2	Artificial joint (2)	CNS (1)/2	24.0, 26.3
7	No	Non.	Non.	Suggestive	Yes	ND	(0)/2	Screw (1), peri-implant tissue (2)	(0)/3	30.0, 30.7, 24.3
8	No	Non.	Non.	Confirmed	No	Negative	(0)/4	Artificial joint	(0)/1	25.6
9	No	Non.	Non.	Confirmed	No	ND	ND	Screw	(0)/1	25.9
10	No	Non.	Non.	Confirmed	No	ND	(0)/3	Artificial joint	(0)/1	28.2
11	No	Non.	Non.	Confirmed	No	ND	(0)/5	Artificial joint (4)	(0)/4	30.8, 28.2, 30.9, 30.7
12	No	Non.	Non.	Confirmed	No	Negative	ND	Artificial joint	(0)/1	28.4
13	No	Non.	Non.	Confirmed	No	Negative	(0)/3	Artificial joint	(0)/1	29.4
14	No	Equivocal	Non.	Confirmed	Yes	Negative	CNS (1)/4	Artificial joint	(0)/1	29.4
15	No	Non.	Non.	Confirmed	No	Negative	(0)/5	Plate	(0)/1	31.7

^*a*^ MSIS/ICM, Musculoskeletal Infection Society/International Consensus Meeting; PJI, periprosthetic joint infection; FRI, fracture-related infection; qPCR Cq, quantification cycle; NGS, next-generation sequencing; ND, not done; CNS, coagulase-negative *Staphylococcus* sp. Note: genus-level pathogens identified in the table (e.g., *Streptococcus*, *Corynebacterium*) are presented in italics in accordance with international microbiological standards.

^*b*^ The ratio represents the number of positive specimens relative to the total number of samples collected.

**TABLE 8:** Comparison of conventional culture results and NGS taxonomic assignments in culture-positive infected cases using the R package decontam^*a*^

Case no. (Table 2)	Age	Sex	CRP (mg/L)	Reason for surgery	Joint	Received antibiotics	Aspiration joint fluid (preoperative) culture	Peri-implant culture	Sonicated implant	Sonicate fluid (positive number)	Concordance with NGS results
1	64	F	276	PJI	Hip	+	*Streptococcus* sp.	Negative	Artificial joint; stem, UHMWPE fiber cable	*Streptococcus* sp. (2)	Exact
3	55	M	92	FRI	Hip	−	CNS	CNS	Nail	CNS	Partial
4	83	M	11	PJI	Hip	−	–	Negative	Artificial joint (2); cup, stem	*Coryne*. sp. (2)	None
6	70	M	30	PJI	Knee	+	CNS	Negative	Artificial joint; tibial insert	CNS	Partial

^*a*^ CRP, C-reactive protein; +, presence; −, absence; UHMWPE fiber cable, ultrahigh-molecular-weight polyethylene fiber cable; CNS, coagulase-negative *Staphylococcus* sp.; *Coryne*. sp., Gram-positive bacillus resembling *Corynebacterium* sp.

**TABLE 9:** Clinical performance report and comparison of diagnostic tests in the next-generation sequencing^*a*^^,^^*f*^

Test	Cases (*N* = 15)		Sensitivity	Specificity	Positive predictive value	Negative predictive value
	OIAI	Non–infected				
	No. of patients with positive specimens^*b*^		(95% CI)			
Conventional culture						
Joint-fluid, Peri-implant tissue culture						
≥1 Positive culture	3/4	1/11	75 (22-99)	91 (57-100)	75 (22-99)	91 (57-100)
≥2 Positive cultures^*b*^	1/4	0/11	25 (1-78)	100 (68-100)	77 (49-95)	79 (49-94)
Sonicate fluid culture^*c*^						
≥1 Positive culture	4/4	0/11	100 (40-100)	100 (68-100)	100 (40-100)	100 (68-100)
≥2 Positive cultures^*b*^	2/4	0/11	50 (9-91)	100 (68-100)	100 (20-100)	85 (54-97)
Sonicate fluied PCR ^*d*^						
≥1 Positive PCR	4/4	3/11	100 (40-100)	73 (39-93)	57 (20-88)	100 (60-100)
≥2 Positive PCR	1/4	0/11	25 (1-78)	100 (68-100)	79 (49-95)	79 (49-94)
Sonicate fluied PCR with NGS^*e*^						
≥1 Positive PCR with NGS	3/4	0/11	75 (22-99)	100 (68-100)	100 (31-100)	92 (60-100)
≥2 Positive PCR with NGS	1/4	0/11	25 (1-78)	100 (68-100)	77 (49-95)	79 (49-94)

^*a*^ Clinical Calculator 1 was used for calculation of sensitivity, specificity, positive and negative predictive value, and confidence intervals (18, 29).

^*b*^ The number of cultures positive for the same microorganism is given.

^*c*^ Sonicate-fluid culture with centrifugation (≥1 CFU/plate); the number of colony-forming units (CFUs) per agar plate growing on either aerobic or anaerobic plates (whichever yielded higher counts) is given.

^*d*^ Positive PCR was defined as qPCR with a Cq value of ≤25.4.

^*e*^ Positive PCR with NGS; a single positive PCR result with NGS occurs when the same bacteria are detected by both PCR and NGS in preoperative and intraoperative culture tests. Alternatively, a positive result occurs when the same bacteria are detected in two or more NGS samples, even if they are not present in the culture tests.

^*f*^ NGS, next-generation sequencing.

The surgical specimen culture results for all patients are summarized in Table 7. In all four infected cases, SFCs were positive. Notably, in three of these cases, bacteria were detected exclusively in the SFCs, whereas in the remaining case, pathogens were identified in both peri-implant tissue and SFCs. Among the cases with suspected infection, qPCR-positive results were observed in two patients presenting with hip prosthesis loosening. Furthermore, in a non-infected patient who underwent primary total hip arthroplasty for post-traumatic osteoarthritis, a qPCR-positive result was observed in the residual implant samples from the initial trauma surgery.

Preoperative NGS analysis revealed multiple bacterial species in one case of suspected septic loosening, whereas *Cutibacterium* was detected in another case. Crucially, in a patient with post-traumatic osteoarthritis in whom infection was not suspected at the time of the primary THA, PJI developed 2 years after surgery during the 3-year clinical follow-up. Retrospective analysis confirmed that the *Staphylococcus* species detected at the onset of PJI had already been identified in the baseline NGS results from the initial surgery.

### Bacterial dominance and potential contaminants in low-biomass samples

The complete NGS results for all clinical specimens are detailed in Tables S2 and S3. Using the predefined cutoff value of Cq ≤ 25.4 (corresponding to 10⁵ CFU/mL of *E. coli*), the clinical samples were stratified into two groups: low-biomass and high-biomass. To compare the microbial profiles between these groups, the top ten taxa were selected based on their relative abundances at the order, family, and genus levels (see Table S3). In the low-biomass samples, *Streptococcus* (rank 1) and *Cutibacterium* (rank 2) accounted for disproportionately large fractions of the sequencing reads, suggesting that these taxa represent typical background contamination under low-biomass conditions.

### Concordance between SFC and NGS for pathogen detection

Among the six culture-positive SFCs, the same bacterial genus was identified via NGS as the dominant taxon in four samples across three cases (cases 1, 3, and 6; Table 8). Conversely, in the remaining two culture-positive samples, discrepancies were observed between conventional culture and post-decontamination NGS results; specifically, conventional cultures identified *Corynebacterium*, whereas NGS identified *Cutibacterium* as the dominant genus (case 4; Table 8).

### NGS analysis of qPCR-positive cases with occult infections

**Fig 6:**
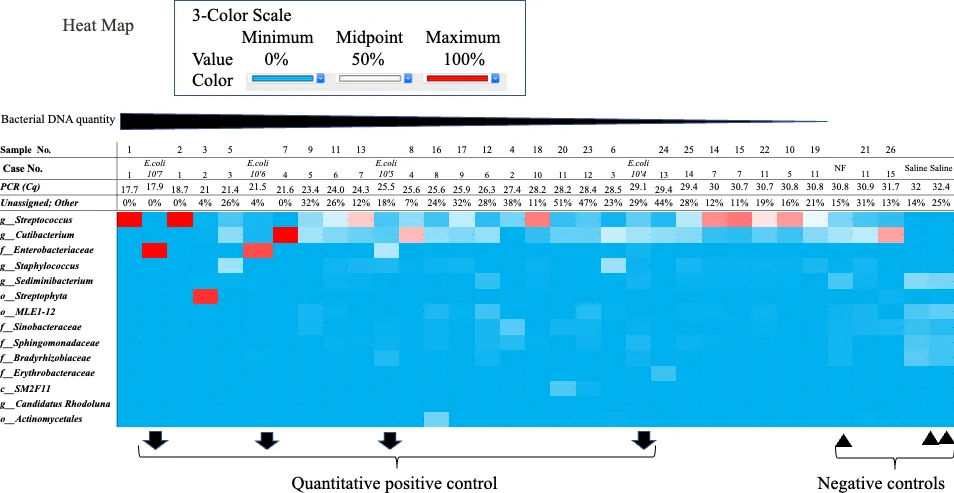
Heatmap visualization of microbial profiles and bacterial DNA quantities. The heatmap illustrates the relative taxonomic abundance across clinical specimens and control samples. Relative abundance values are color-coded based on a three-color scale: minimum (0%, blue), midpoint (50%, white), and maximum (100%, red). Samples are ordered on the horizontal axis by bacterial DNA quantity corresponding to the Cq values. On the vertical axis, bacterial taxa are listed in descending order of their cumulative relative abundance across all sequencing reads, reflecting operational taxonomic classifications. Black arrows indicate the quantitative positive controls (*Escherichia coli* dilutions from 10^7^ to 10^4^ CFU/mL), while the black triangle and double triangles denote the NF water and sterile saline negative controls, respectively.

We conducted a detailed taxonomic analysis at the genus level and below using a three-step framework: (i) genus-level classification, (ii) pre-decontamination profiling (excluding chloroplast and plant-related lineages), and (iii) post-decontamination analysis using the *decontam* algorithm. In one culture-negative case (case 2, sample 3 [see Fig. S2]), qPCR and initial NGS analysis identified *Streptophyta*. However, after filtering out these chloroplast- and plant-derived contaminant sequences, *decontam* analysis excluded *Streptophyta* and revealed a polymicrobial profile; the most abundant genus was *Streptococcus* (20%), followed by *Cutibacterium* (17%) and *Anaerococcus* (14%) (see Table S5). Other identified genera included *Paracoccus* (11%), *Staphylococcus* (7%), *Burkholderia* (7%), and *Candidatus Rhodoluna* (5%), with minor taxa accounting for the remaining 19% of the total reads (see Table S5). In the two cases described below, removing these contaminant signals and performing *decontam* analysis did not alter the detected bacterial taxa. Instead, the relative abundance of each remaining taxon increased (see Table S5). For instance, in another culture-negative, qPCR-positive sample from case 5 (see Table S6), NGS identified *Cutibacterium* (32% pre- and 36% post-decontamination) and *Streptococcus* (28% vs 32%, respectively) (see Table S6). Similarly, a primary total hip arthroplasty (THA) case (case 7) for post-traumatic osteoarthritis (see Fig. 6; Table S7) yielded a qPCR-positive sample (Cq = 24.3, sample 13, Table S7) that contained *Streptococcus* (60% pre- and 63% post-decontamination), *Cutibacterium* (20% vs 22%), and *Staphylococcus* (5% vs 5%).

## DISCUSSION

A key finding of this study is the strong technical reliability and reproducibility of our 16S rRNA gene qPCR protocol, which serves as a crucial foundation for accurate downstream NGS analysis. As demonstrated by our validation using serial *E. coli* dilutions, Cq measurements exhibited minimal variability, with an ICC [1,1] of 0.961 (see Fig. 3). This high level of reproducibility strongly aligns with our previous methodological validation, which reported near-perfect reliability with a single-measure ICC of 0.991 and an average-measure ICC of 0.998 (14). The statistical significance of these values, further supported by the robust F-test results (F = 441.520, *P* < 0.001), underscores that our molecular assay yields highly consistent results with negligible technical noise across different bacterial biomass levels. In the context of microbiome-based diagnostics, establishing such rigorous intra-assay consistency is paramount. High-throughput sequencing platforms, particularly when applied to low-biomass clinical specimens like orthopedic sonication fluids, are highly susceptible to false positives arising from background noise or reagent contamination (12, 30). By confirming that our qPCR pre-screening method delivers tightly reproducible Cq values, we demonstrate its utility not only as a standalone diagnostic aid but also as an essential quality-control filter prior to NGS testing (10). Utilizing this protocol as a screening tool allows clinicians to objectively assess bacterial load thresholds, ensuring that only high-confidence samples proceed to expensive sequencing workflows, thereby enhancing both diagnostic accuracy and cost-effectiveness in clinical practice.

### Clinical-based Cq cutoff value obtained from statistical analysis

The ROC curve (see Fig. 4) exhibited an area under the curve (AUC) of 0.820 (95% CI: 0.735–0.905), suggesting that this genetic test possesses good discriminatory ability for the target disease. The result was statistically significant (*P* < 0.001), rejecting the null hypothesis that the true AUC is 0.5. The ROC curve demonstrates a favorable balance between sensitivity and specificity across different thresholds, suggesting that qPCR is a useful tool for distinguishing between positive and negative cases in this setting. Although variations in amplification efficiency in samples near the limit of detection can affect the sensitivity–specificity trade-off due to the nature of qPCR, the fact that the lower limit of the CI is 0.735 indicates a high level of reliability for clinical implementation.

### Characteristics of sequencing results near the quantitative limit and the impact of biomass on microbial profiles

**Fig 7:**
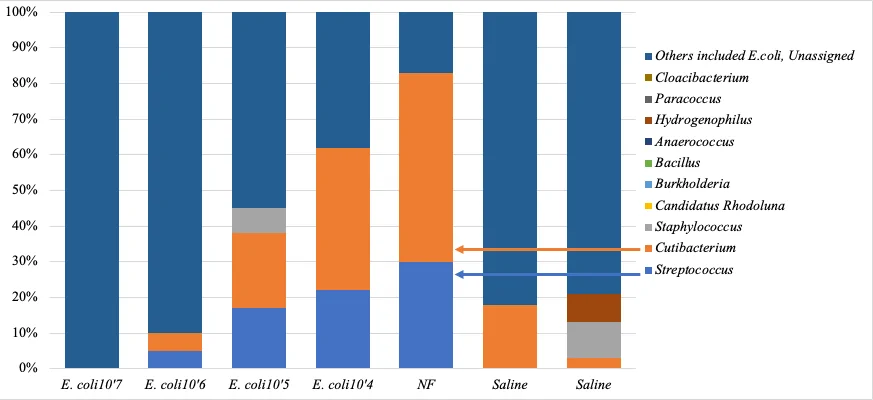
Tipping point of microbial profile convergence after processing with the *decontam* package. The stacked bar chart illustrates the relative taxonomic abundance of the top ten bacterial genera across serially diluted *Escherichia coli* positive controls and negative controls after processing with the *decontam* package. Samples are arranged on the horizontal axis in order of decreasing bacterial biomass. The composition of the highly diluted positive controls progressively transitions and converges with the background contamination profiles observed in the NF water and sterile saline negative controls.

Regarding bacterial taxa other than *E. coli*, the analytical outcomes after applying *decontam* and filtering out chloroplast-associated reads revealed critical insights into low-biomass sequencing behavior. At a dilution concentration of 10^4^ CFU/mL, the sequencing results were nearly identical to the NGS profiles of NF water samples. As the bacterial concentration gradually decreased from 10^7^ CFU/mL, the taxonomic composition showed a progressive trend toward mimicking the NF water sequencing data (see Fig. 7). This finding demonstrates that under low-biomass conditions, even when sterile physiological saline is utilized for sample dilution, the resulting profile inherently converges with the background noise obtained from NF water within the NGS processing workflow (see Table S5). The relative abundance of bacterial taxa was highly dependent on the initial DNA concentration of the samples. As shown in Fig. 7, samples with low Cq values (reflecting high DNA concentrations, or high-biomass conditions) were heavily dominated by the target biological signal. However, a progressive increase in the proportions of *Cutibacterium* and *Streptococcus* was observed as the DNA concentration decreased (corresponding to increasing Cq values). This inverse correlation strongly suggests that low-biomass samples are particularly susceptible to background contamination, even after computational decontamination procedures. Our results highlight the inherent challenges of interpreting microbial profiles in low-biomass environments, indicating that when the target DNA concentration falls below a critical threshold, the true biological signal becomes indistinguishable from background noise. This necessitates a cautious interpretation of taxonomic data in high-Cq samples. While high-biomass samples maintained a clear and reliable profile, the prominent emergence of *Streptococcus* and *Cutibacterium* in the highly diluted series (see Fig. 7) suggests that current decontamination methods may not completely eliminate reagent-derived or laboratory-acquired DNA. Taken together, these findings underscore the paramount importance of including negative controls (see Fig. 6 and 7) and implementing concentration-based filtering thresholds in metagenomic pipelines (see Table 9).

### Bacterial dominance, contamination risk, and the necessity of a Cq-based cutoff in low-biomass environments

Moderate correlations were observed between Cq values and the relative abundances of *Sediminibacterium*, MLE1-12, and *Sinobacteraceae* among the top ten most abundant bacterial taxa identified by NGS in the implant sonicated fluid samples. A weak correlation was also observed for *Sphingomonadaceae* (see Table S3). Consistent with our previous analyses demonstrating a positive correlation between Cq values and the relative abundance of specific background bacteria, these taxa were classified as likely contaminants and systematically removed using *decontam* (see Table S3). Furthermore, seven of the top ten taxa were consistently detected in both the high- and low-biomass groups (see Table S3). Notably, *Streptococcus* and *Cutibacterium* were observed at high frequencies, even in samples with exceptionally low bacterial DNA concentrations. This finding mirrors the contamination profiles observed in our negative controls (see Fig. 6). Because these two genera are well-documented opportunistic pathogens capable of causing orthopedic infections, they must be rigorously differentiated from background contamination. However, their high prevalence in low-biomass specimens makes it challenging to determine whether they represent true causative pathogens or experimental artifacts based on NGS data alone. These findings highlight a critical limitation of NGS in low-biomass samples: the potential distortion of microbial community compositions by contaminating background bacteria (9, 10). Such contamination can obscure true biological signals, leading to the misinterpretation of high-throughput sequencing results. Our data indicate that even after applying computational contamination removal tools, the resolution remains incomplete. While these bioinformatic tools are highly effective for high-biomass samples, they do not necessarily elucidate the true microbial profile under low-biomass conditions. To address this technical limitation, establishing a Cq-based quality control cutoff is essential. In this study, a concentration-dependent tipping point was identified between Cq values of 25.5 (10^4^–10^5^ CFU/mL) and 26.4 (10^4^ CFU/mL), at which the microbial composition shifted from target-organism dominance to contamination dominance. Consequently, NGS data obtained from samples yielding Cq values ≥ 25.5 (10^4^–10^5^ CFU/mL) likely reflect experimental artifacts rather than the actual *in vivo* microbial composition of the specimen. Our results also revealed a critical discrepancy between the clinical qPCR diagnostic threshold and the analytical detection limit of NGS. At the cutoff value utilized to infer infection via qPCR, the bacterial concentration falls within the low-biomass range, resulting in compromised accuracy when evaluated by NGS (see Fig. 5). Therefore, integrating complementary quantitative methods, such as the present qPCR assay, is essential to safeguarding the accuracy of NGS-based bacterial analyses in clinical settings (see Table 9).

### Concordance between culture and NGS methods for pathogen identification

In infected cases, four of the six culture test results were fully consistent with the post-decontamination NGS outcomes (see Table 8). One patient (case 4) presented with two unmatched results: conventional cultures identified *Corynebacterium* spp., whereas NGS identified *Cutibacterium* spp. as the dominant genus (sample 7; Fig. 6; Fig. S2). This discrepancy is likely attributable to a conventional, culture-based identification error rather than an analytical artifact. Several lines of evidence support this interpretation. First, previous studies utilizing identical 16S rRNA universal primers (8F–338R) confirmed the effective amplification and detection of both *Corynebacterium* and *Cutibacterium* lineages (24). Second, *Cutibacterium* was successfully detected in our baseline validation at the expected frequency (see Table S1). Third, the present NGS workflow has been previously validated for the successful identification of *Cutibacterium* in cases of chronic osteomyelitis (13). Fourth, Corynebacterium can be robustly sequenced with primers targeting the same V1–V2 variable regions of the bacterial 16S rRNA gene using the Ion Torrent PGM platform (25). Overall, these findings strongly suggest that the observed discrepancy did not arise from limitations in primer design, sequencing hardware, or bioinformatic pipelines. Instead, the misidentification likely stems from the inherent limitations of conventional phenotyping. The biochemical characteristics of *Corynebacterium* species are notoriously difficult to confirm using routine bacteriological testing, making accurate species-level identification highly challenging. Previous research has underscored the systemic difficulty of identifying coryneform bacteria within clinical microbiology laboratories using traditional phenotypic assays (31). High-throughput sequencing can effectively overcome these diagnostic hurdles by providing reliable, sequence-based classification of fastidious bacteria with overlapping biochemical or phenotypic characteristics, which are frequently misclassified or rendered unidentifiable by conventional culture-based systems.

### NGS analysis of qPCR-positive cases with occult infections

In case 2 (see Fig. S3), microbiome analysis of the loosened prosthetic hip joint after applying the *decontam* package revealed a highly diverse bacterial composition supported by a high bacterial load. Although the prominent background signals made it challenging to definitively evaluate the pathogenic roles of *Streptococcus* and *Cutibacterium*, the results suggest that multiple bacterial species, including *Staphylococcus*, may have colonized the implant surface. Since these samples met validated quantification thresholds, these bacteria should not be immediately dismissed as technical artifacts. The identified *Streptococcus* lineages are dominant species in the human oral microbiome, and these molecular findings suggest that asymptomatic colonization by the oral commensal microbiota may contribute to the pathogenesis of delayed implant loosening through low-level, multi-species biofilm structures, rather than sterile mechanical damage (32–34).

Similarly, in case 5 (see Fig. 6; Fig. S4), a one-stage revision THA was performed for suspected septic loosening. Although all conventional cultures were negative, one of the two intraoperative samples was qPCR-positive. NGS analysis of this high-biomass sample (sample 9; Table S6) identified *Cutibacterium* spp. (32%) and *Streptococcus* spp. (28%). Notably, the microbial composition shifted markedly between samples with differing bacterial DNA content (samples 9 and 10; Table S6). Specifically, the relative abundance of *Streptococcus* increased from 28% to 70% as the bacterial DNA content decreased, suggesting that *Streptococcus* represents a background contaminant in low-biomass environments. Conversely, the abundance of *Cutibacterium* decreased from 32% to 12% alongside declining bacterial DNA content, which aligns with the hypothesis that true biological signals diminish concurrently with reductions in total bacterial DNA (13, 14). For this reason, *Cutibacterium* was considered the true causative pathogen. Furthermore, post-decontamination profiling revealed *Burkholderia* at 3% in sample 9, and although the proportions of *Cutibacterium* and *Streptococcus* increased slightly, the overall biomass-dependent trends remained unaltered. In contrast, the low-biomass sample (sample 10; Table S6) exhibited a pattern highly prone to contamination. This contrast illustrates the complex, biomass-dependent difficulty of interpreting low-biomass specimens, rather than a simple binary distinction between pathogen and contaminant.

A third patient (case 7; Fig. S5, Table S7) underwent primary THA for post-traumatic osteoarthritis, which was initially managed as a non-infected case. Crucially, 2 years after the primary THA, this patient developed a delayed PJI requiring a two-stage revision procedure, during which *Staphylococcus* was identified as the causative pathogen via culture. At the time of the primary surgery, both histopathology and conventional cultures were negative; however, a single screw-derived sample yielded a positive qPCR result (sample 13; Table S7, Cq = 24.3), with a Cq value below the NGS-derived cutoff of 25.4, indicating a bacterial DNA load above the corresponding threshold. Baseline NGS analysis of this sample identified *Streptococcus* spp. (60%), *Cutibacterium* spp. (21%), and *Staphylococcus* spp. (5%) (see Table S7). Although sample 13 was classified as high-biomass based on the Cq threshold, its taxonomic profile partially overlapped with the background noise of the NF water negative control, unlike the true negative samples (samples 14 and 15). This finding suggests that even samples satisfying the qPCR-based Cq threshold cannot be interpreted in isolation; instead, they must be comprehensively evaluated alongside taxonomic plausibility, contamination-aware filtering, and clinical context.

Given the patient’s history of severe post-traumatic osteoarthritis following an acetabular fracture, the subclinical presence of bacteria on the implants was not entirely unexpected. A systematic review assessing THA outcomes following acetabular fractures reported that the risk of infection is substantially higher than in conventional hip arthroplasties, particularly in patients with multiple prior surgeries and retained internal fixation hardware (35). Furthermore, Knabl et al. demonstrated asymptomatic bacterial colonization of osteosynthesis implants in healthy patients without any clinical or laboratory signs of infection (36). Similar colonization phenomena have been documented in non-orthopedic fields, such as the subclinical colonization of cardiac electrophysiological devices (37).

These observations underscore the extreme complexity of distinguishing true low-abundance biological signals from environmental contaminants, especially in post-traumatic reconstructions. Among the three patients in our cohort, two were managed intraoperatively under the clinical suspicion of infection and achieved uneventful long-term outcomes, whereas the remaining patient (case 7), in whom infection was not initially suspected or treated, subsequently required major revision surgery for delayed PJI. Although we cannot definitively conclude whether the baseline NGS signals directly reflected an active initial infection, the presence of qPCR-positive samples in all three cases cannot be ignored. The fact that the patient who did not receive proactive antimicrobial management later developed an indisputable PJI supports the potential clinical utility of our molecular screening framework to detect occult pathogens before they manifest clinically.

### Addressing false positives and contamination: past challenges and future perspectives

A fundamental challenge in interpreting NGS data is that the methodology provides relative taxonomic proportions rather than absolute bacterial quantification. When a specimen contains a high bacterial DNA concentration, the resulting NGS profile is typically characterized by a high proportion of reads corresponding to a dominant pathogen. Indeed, in cases of overt bacterial infection, one or two bacterial genera usually dominate the sequencing output. In contrast, specimens with low bacterial abundance or those from non-infected cases frequently yield polymicrobial NGS profiles composed of multiple genera, significantly complicating clinical interpretation.

Consequently, these methodological limitations have posed a long-standing barrier to the reliable interpretation of NGS findings. To date, detailed sequencing analyses focusing specifically on low-biomass specimens have not been rigorously conducted within the field of orthopedics. Although the sample size of the present study is relatively small, to our knowledge, this is the first orthopedic NGS investigation to specifically address and systematically stratify low-biomass samples using rigorous negative controls. Therefore, our findings are expected to serve as a foundational benchmark for future NGS research evaluating the implant-associated microbiota.

### Limitations of the study

This study has several methodological limitations that should be considered when interpreting our findings. First, the clinical Cq values and established thresholds reported herein are inherently subject to inter-facility and inter-instrument variations, meaning they cannot be directly generalized without local calibration. The precise reliability and reproducibility of these absolute Cq metrics must be independently validated against characterized bacterial loads at individual testing facilities. Second, our total sample size was relatively limited, as the high-throughput NGS data were derived from a single sequencing run encompassing a specific patient cohort. To refine the operational limits of bacterial detection under low-biomass conditions, even stricter quantitative and qualitative quality control criteria may need to be implemented in larger prospective studies. Furthermore, extensive validation of identical clinical specimens under diverse experimental conditions remains necessary. This includes evaluations across different laboratories and on alternative sequencing platforms—such as short-read technologies like Illumina or long-read platforms like Oxford Nanopore vs the Ion Torrent platform utilized here. Comprehensive monitoring and multi-center cross-validation are required to further validate the universal robustness and reproducibility of our proposed diagnostic framework.

### Conclusion

In conclusion, this study supports our hypothesis that NGS may support bacterial identification with negligible background noise when sample selection is guided by a designated Cq threshold. In overtly infected cases, NGS-based identification demonstrated high concordance with conventional cultures, while simultaneously exposing potential classification errors inherent to traditional phenotypic methods. Furthermore, our molecular workflow highlighted the capacity of NGS to resolve occult infections in culture-negative cases. However, because high-throughput sequencing provides relative taxonomic proportions rather than absolute quantification, distinguishing likely causative pathogens from environmental or reagent contaminants remains challenging in low-biomass environments. Our findings underscore that interpreting microbiome data from low-biomass specimens requires exceptional caution, as computational decontamination tools cannot completely eliminate low-level background artifacts. To address this technical hurdle, we demonstrated that integrating qPCR with NGS and applying a stringent analytical Cq cutoff effectively mitigates false-positive signals. Therefore, when utilizing NGS for the diagnosis of PJI, incorporating quantitative assessments alongside rigorous negative and positive controls is paramount. For cases with borderline or inconclusive sequencing profiles, long-term clinical monitoring remains indispensable for definitive determination. Ultimately, larger multi-institutional prospective studies are warranted to refine these diagnostic thresholds, enhance NGS resolution, and firmly establish its clinical applications in orthopedic practice.

## Supplementary Material

Reviewer comments

## Data Availability

The data sets generated and analyzed during the current study are available from the corresponding author upon reasonable request.

## SUPPLEMENTAL MATERIAL

Supplemental figures and tables spectrum.04128-25-s0001.pdf Figures S1 to S5 and Tables S1 to S7.

OPEN PEER REVIEW reviewer-comments.pdf An accounting of the reviewer comments and feedback.

ASM does not own the copyrights to Supplemental Material that may be linked to, or accessed through, an article. The authors have granted ASM a non-exclusive, world-wide license to publish the Supplemental Material files. Please contact the corresponding author directly for reuse.
