# Supplementary material for: The diagnostic potential of combined quantitative polymerase chain reaction and next-generation sequencing using the same primers for periprosthetic joint infection
Source: Microbiol Spectr. 2026 Jul 28;14(9):e04128-25. doi: 10.1128/spectrum.04128-25 (PMC13532281; doi:10.1128/spectrum.04128-25)
Supplement: Supplementary file 1 [file reviewer-comments.pdf]

AMERICAN SOCIETY FOR MICROBIOLOGY | Microbiology Spectrum

The Diagnostic Potential of Combined Quantitative Polymerase Chain Reaction and Next-Generation Sequencing Using the Same Primers for Periprosthetic Joint Infection

Narumi Ueda, Jun Inoue, Kazuyuki Okuda, and Hirokazu Iida

Corresponding Author(s): Narumi Ueda, Nihon Sekijujisha Wakayama Iryo Center

Review Timeline:

Submission Date:	December 22, 2025
Editorial Decision:	February 24, 2026
Revision Received:	May 28, 2026
Accepted:	June 8, 2026

Editor: Takeaki Wajima

Reviewer(s): Disclosure of reviewer identity is with reference to reviewer comments included in decision letter(s). The following individuals involved in review of your submission have agreed to reveal their identity: Lorenzo Drago (Reviewer #1); Emi Tanaka (Reviewer #2)

Transaction Report:

(Note: With the exception of the correction of typographical or spelling errors that could be a source of ambiguity, letters and reports are not edited. The original formatting of letters and referee reports may not be reflected in this compilation.)

DOI: https://doi.org/10.1128/spectrum.04128-25

Re: Spectrum04128-25 (The Diagnostic Potential of Combined Quantitative Polymerase Chain Reaction and Next-Generation Sequencing Using the Same Primers for Periprosthetic Joint Infection)

Dear Dr. Narumi Ueda:

Thank you for the privilege of reviewing your work. Below you will find my comments, instructions from the Spectrum editorial office, and the reviewer comments.

Both reviewers agree that the study is conceptually valuable but requires substantial improvement before it can support robust conclusions. Key concerns include the generalizability of the Cq cutoff, contamination control, lack of statistical analyses, and issues in structure and interpretation.

Please return the manuscript within 60 days; if you cannot complete the modification within this time period, please contact me. If you do not wish to modify the manuscript and prefer to submit it to another journal, notify me immediately so that the manuscript may be formally withdrawn from consideration by Spectrum.

Revision Guidelines
To submit your modified manuscript, log into the submission site at https://spectrum.msubmit.net/cgi-bin/main.plex. Go to Author Tasks and click the appropriate manuscript title to begin. The information you entered when you first submitted the paper will be displayed; update this as necessary. Note the following requirements:

• Upload point-by-point responses to the issues raised by the reviewers in a file named "Response to Reviewers," NOT in your cover letter.
• Upload a compare copy of the manuscript (without figures) as a "Marked-Up Manuscript" file.
• Upload a clean .DOC/.DOCX version of the revised manuscript and remove the previous version.
• Each figure must be uploaded as a separate, editable, high-resolution file (TIFF or EPS preferred), and any multipanel figures must be assembled into one file.
• Any supplemental material intended for posting by ASM should be uploaded with their legends separate from the main manuscript. You can combine all supplemental material into one file (preferred) or split it into a maximum of 10 files with all associated legends included.

For complete guidelines on revision requirements, see our Submission and Review Process webpage. Submission of a paper that does not conform to guidelines may delay acceptance of your manuscript.

Data availability: ASM policy requires that data be available to the public upon online posting of the article, so please verify all links to sequence records, if present, and make sure that each number retrieves the full record of the data. If a new accession number is not linked or a link is broken, provide Spectrum production staff with the correct URL for the record. If the accession numbers for new data are not publicly accessible before the expected online posting of the article, publication may be delayed; please contact production staff (Spectrum@asmusa.org) immediately with the expected release date.

Publication Fees: For information on publication fees and which article types are subject to charges, visit our website. If your manuscript is accepted for publication and any fees apply, you will be contacted separately about payment during the production process; please follow the instructions in that e-mail. Arrangements for payment must be made before your article is published.

ASM Membership: Corresponding authors may join or renew ASM membership to obtain discounts on publication fees. Need to upgrade your membership level? Please contact Customer Service at Service@asmusa.org.

The ASM Journals program strives for constant improvement in our submission and publication process. Please tell us how we can improve your experience by taking this quick Author Survey.

Thank you for submitting your paper to Spectrum.

Sincerely,
Takeaki Wajima
Editor
Microbiology Spectrum

Reviewer #1 (Comments for the Author):

The manuscript con be considered an interesting proof-of-concept showing that adding 16S qPCR (Cq values) helps interpret low-biomass 16S NGS from sonicate fluid in suspected PJI, but the small sample size and contamination/threshold handling limit

the strength of clinical conclusions. My recommendation is Major revision.

It debates a clinically relevant problem (PJI diagnosis) using sonicate fluid and a pragmatic "qPCR + NGS with same primers" approach. However: a clear demonstration that low bacterial load and NGS profiles become contamination-dominated should be motivated with quantitative context and parameters.

Major issues to address

Cq cutoff (25.5) generalizability: Derived from an E. coli dilution series; may not translate to typical PJI organisms and is complicated by noted primer bias. Provide qPCR performance metrics and sensitivity analysis across cutoffs. Most bacteria responsible of PJIs are gram positives.

Contamination control: Negative controls are included, but decontamination is mostly descriptive (e.g., relative abundance thresholds). Use/describe a reproducible contaminant-identification strategy and show results with/without it.

Taxonomic interpretation: The "loosening/oral flora" inference is weak because a key sample is dominated by Streptophyta, which often reflects chloroplast/plant-related signal in 16S datasets. Confirm sequences and clarify filtering.

Clinical performance reporting: Provide sensitivity/specificity/ PPV/NPV for qPCR, NGS, and the combined strategy using your infection definition; account for multiple samples per patient.

Bioinformatics choices: Justify >3% "significant genus" cutoff, 10k rarefaction, OTU97%, and Greengenes use; discuss how these choices affect pathogen detection.

Minor points

Update/standardize terminology (e.g., Propionibacterium/Cutibacterium).

Clarify sample accounting (patients vs samples vs sequenced) and add a simple flow table.

Reviewer #2 (Comments for the Author):

This manuscript addresses an important and increasingly relevant topic in the diagnosis of periprosthetic joint infection (PJI): the integration of quantitative PCR (qPCR) and 16S rRNA amplicon sequencing using identical primers, with a focus on bacterial biomass and contamination in low biomass samples. The study design is thoughtful, the use of multiple controls is commendable, and the clinical relevance is high. However, several key issues related to structure, statistical rigor, and interpretation must be addressed.

Major Comments

1. Clarity and focus of the Introduction

The Introduction is informative but overly long and diffuse. Multiple themes-low-biomass challenges, contamination, primer bias, limitations of previous orthopedic NGS studies, and the study's aims-are interwoven in a way that obscures the core rationale. Please restructure this section and present a concise paragraph outlining the specific aims of the study early in the Introduction.

2. Statistical analysis

Although the experimental work is robust, the manuscript lacks statistical evaluations. For example, no statistical comparison between high- and low-biomass groups, no confidence intervals for determining the Cq cutoff, no evaluation of contamination taxa using available computational or statistical tools. Please include at least basic statistical analyses (e.g., Mann-Whitney U tests, Wilcoxon signed-rank tests, or measures of variability for control samples).

3. Justification and generalizability of the Cq = 25.5 cutoff

The cutoff is experimentally derived using E. coli, which is reasonable, but the manuscript does not discuss its applicability to other taxa or primer bias associated with the V1-V2 region. Please elaborate on the limitations of using a single organism to establish a universal threshold.

4. Interpretation of low-biomass findings

Some statements-particularly those regarding Streptophyta and oral bacteria potentially contributing to implant loosening-are too strong given the possibility of contamination and the constraints of 16S methods.

5. Figures

The manuscript contains numerous figures, some of which are highly detailed. This makes it difficult for readers to identify the main findings. Please consider moving detailed or case-specific figures (e.g., full NGS histograms for all samples) to the

Supplementary Materials.

RESPONSE TO THE REVIEWER'S COMMENTS

Manuscript ID: Spectrum04128-25

Title: The Diagnostic Potential of Combined Quantitative Polymerase Chain Reaction and Next-Generation Sequencing Using the Same Primers for Periprosthetic Joint Infection)

Dear Editor and Reviewer,

We would like to express our sincere gratitude to the reviewer for the time, effort, and constructive feedback dedicated to our manuscript. We highly appreciate the positive evaluation.
In accordance with the reviewer's excellent recommendation, we have thoroughly expanded the Discussion section to explicitly address the clinical utility, consensus strategies, and diagnostic trade-offs associated with combining conventional cultures and the qPCR assay.
In addition, new analysis was added NGS study.
Below is our point-by-point response to the reviewer's comment, including the exact text added to the revised manuscript.

Reviewer #1 (Comments to the Author):
Comment:
The manuscript con be considered an interesting proof-of-concept showing that adding 16S qPCR (Cq values) helps interpret low-biomass 16S NGS from sonicate fluid in suspected PJI, but the small sample size and contamination/threshold handling limit the strength of clinical conclusions. My recommendation is Major revision.
It debates a clinically relevant problem (PJI diagnosis) using sonicate fluid and a pragmatic "qPCR + NGS with same primers" approach. However: a clear demonstration that low bacterial load and NGS profiles become contamination-dominated should be motivated with quantitative context and parameters.

Response:
We sincerely thank the reviewer for evaluating our work as an "interesting proof-of-concept" and highlighting the clinical relevance of our pragmatic "qPCR + NGS using the same primers" approach. We agree that a rigorous, parameter-driven framework is required to definitively demonstrate the threshold where low bacterial loads become dominated by background contamination.
To address this crucial point, we have significantly reinforced the quantitative context of our study by implementing the following revisions:
1. Clear Parameter-Based Definition of the Tipping Point: We have explicitly contextualized the sequencing profiles of our serial *E. coli* dilutions and clinical specimens using strict qPCR Cq values. As illustrated in the revised Figure 7, we demonstrated that when the bacterial biomass falls below a critical threshold (Cq = 25.5, corresponding to 10^5 CFU/mL), the target biological signal systematically diminishes, and the NGS profile dynamically converges with the contamination signatures observed in our nuclease-free water and saline negative controls.
2. Integration of Computational Decontamination Metrics: Rather than relying on visual interpretation alone, we have expanded our methodological transparency regarding the R package *decontam*. We explicitly detailed the prevalence-based threshold parameters (systematically evaluated from 0.1 to 0.5, with 0.3 selected as the optimal consensus cutoff) used to mathematically differentiate true pathogens from experimental noise (Supplementary Figure S1).
3. Refined Discussion on Clinical Implementation: We have expanded the Discussion section to address how these quantitative boundaries prevent the misinterpretation of low-biomass data, which has been a long-standing challenge in molecular diagnostics. By pairing the qPCR screening threshold ($Cq \leq 25.4$) with NGS, our workflow provides an objective quality-control filter to safeguard diagnostic specificity.
To further provide clear, quantitative evidence demonstrating that our threshold handling successfully isolates true pathogens from contamination-dominated low-biomass profiles, we have included a rigorous

Clinical Performance Report within our NGS cohort (N=15, summarized in the newly designated Table 9). As shown in Table 9, while standalone sonicate fluid qPCR yielded a high sensitivity (100%) but was prone to low-biomass background noise (reducing standalone specificity to 72.7% for positive PCR), the integration of our NGS-derived cutoff (Cq ≤ 25.4) with the *decontam* algorithm adequately addressed this ambiguity. Crucially, the combined "Sonicate-fluid PCR with NGS" workflow (positive PCR with NGS criterion) achieved a 100% specificity of 100% (0/11 non-infected cases, 95% CI: 68%–100%) and a positive predictive value of 100%.
This parameter-driven performance report demonstrates that our quality-control threshold does not merely filter data arbitrarily; rather, it completely eradicates low-biomass experimental artifacts in non-infected controls while maintaining a robust diagnostic sensitivity to capture true occult infections. We believe this evidence directly reinforces the strength and validity of our clinical conclusions.
We believe that these quantitative parameters have significantly strengthened the rigor of our clinical conclusions and successfully addressed your core methodological concerns.

Major issues to address (1-5)
Comment1:
Cq cutoff (25.5) generalizability: Derived from an *E. coli* dilution series; may not translate to typical PJI organisms and is complicated by noted primer bias. Provide qPCR performance metrics and sensitivity analysis across cutoffs. Most bacteria responsible of PJIs are gram positives.

Response1:
We agree with the reviewer's excellent point that because PJI is predominantly caused by Gram-positive pathogens (such as *Staphylococcus* spp.), validating a Cq cutoff based solely on a Gram-negative Escherichia coli model requires careful justification and explicit quantitative evaluation.
To address this concern rigorously, we have integrated a comprehensive sensitivity analysis and provided detailed qPCR performance metrics across alternative cutoff thresholds in the revised manuscript (now added to the Results section and summarized in the newly compiled Table 9).
Our rationale for selecting the E. coli-derived threshold, along with the newly added metrics, is based on the following three scientific considerations:
1. Conservative Threshold Design based on Primer Bias: As noted in our supplementary methodological validation, our universal 16S rRNA primers (8F–338R) exhibit a known bias where certain Gram-negative lineages display lower amplification efficiency or higher detection limits compared to the highly robust amplification observed for Gram-positive *Staphylococcus*. Consequently, we prioritized setting our NGS analytical cutoff based on the "worst-case scenario" (i.e., the more difficult-to-detect Gram-negative bacteria). Setting the threshold conservatively at Cq ≤ 25.4 ensures that if a Gram-negative pathogen is present, it will not be missed, while simultaneously safeguarding the detection of the more easily amplified Gram-positive PJI pathogens.
2. Standardization of Absolute Quantification: The use of E. coli (ATCC 25922) was necessary to establish a highly reproducible standard curve linked directly to the McFarland turbidity scale for absolute cell-count quantification (104 to 107 CFU/mL), serving as a dependable reference benchmark across independent sequencing runs.
3. Sensitivity Analysis Across Thresholds: In the revised manuscript, we systematically evaluated alternative clinical cutoffs (Cq = 24.5, 25.4, 26.4, and 27.5). As now detailed in the text, lowering the threshold to 24.5 significantly compromised diagnostic sensitivity for low-biomass Gram-positive infections, whereas raising it to 26.4 or 27.5 introduced unacceptably high rates of background contaminant reads. The selected threshold (Cq ≤ 25.4) provided the optimal diagnostic equilibrium to capture true biological signals for both Gram-positive and Gram-negative cohorts while suppressing experimental artifacts. We believe that this expanded quantitative evaluation and sensitivity analysis successfully demonstrates the generalizability of our threshold selection.

Comment2:

Contamination control: Negative controls are included, but decontamination is mostly descriptive (e.g., relative abundance thresholds). Use/describe a reproducible contaminant-identification strategy and show results with/without it.
Taxonomic interpretation: The "loosening/oral flora" inference is weak because a key sample is dominated by *Streptophyta*, which often reflects chloroplast/plant-related signal in 16S datasets. Confirm sequences and clarify filtering.

Response2: We sincerely appreciate the reviewer's rigorous and critical evaluation regarding our contamination control and taxonomic filtering pipeline. We fully agree that transitioning from a descriptive interpretation to a reproducible, parameter-driven framework—and clearly illustrating the profile changes before and after bioinformatic filtering—is essential to validate our clinical inferences.
To address the reviewer's concerns completely, we have clarified our systematic, reproducible multi-step filtering protocol and provided explicit "with vs. without" comparative datasets in the revised manuscript, as detailed below:
1. Reproducible Computational Decontamination Strategy (with/without *decontam*): To address the demand for a reproducible strategy, we expanded our methodology regarding the R package *decontam*. We performed an expanded prevalence-based sensitivity analysis, evaluating thresholds from 0.1 to 0.5. As illustrated in the newly added (Supplementary Figure S1), we demonstrated that the expected top OTUs of our mock community control were preserved up to a threshold of 0.3, whereas true biological signals began to be misclassified as candidate contaminants at thresholds of 0.4 and above. Based on this reproducible algorithmic validation, 0.3 was established as the optimal threshold. Furthermore, to show the explicit impact of this decontamination strategy, we compiled comprehensive "pre- versus post-decontamination" comparative datasets for our key clinical occult-infection cases (Supplementary Tables S5, S6, and S7). These datasets explicitly track the relative abundances of all taxa with and without the application of the *decontam* algorithm.
2. Clarification of Chloroplast/Plant-Derived (*Streptophyta*) Filtering and Biomass-Dependent Interpretation: The reviewer is entirely correct that the predominance of *Streptophyta* represents plant-derived chloroplast noise rather than a true in vivo clinical biofilm signal.
In our initial exploratory NGS analysis of Case 2, *Streptophyta* indeed accounted for 90% of the raw sequencing reads (Supplementary Table S5). To prevent this artifact from distorting our clinical interpretation, we implemented a rigorous three-step taxonomic filtering framework in our bioinformatic pipeline, which has now been explicitly detailed in the Methods and Results sections:
Step 1 (Genus-level classification): Raw profiling capturing all amplified sequences.
Step 2 (Organellar filtering): Systematic exclusion of all chloroplast- and mitochondria-related lineages (specifically filtering out any operational taxonomic units [OTUs] predefined by taxonomy strings containing "c__Chloroplast" or "Mitochondria"). This step successfully removed the overwhelming *Streptophyta* signal.
Step 3 (Prevalence-based *decontam* processing): Algorithmic background removal utilizing the negative references. Importantly, we acknowledge the reviewer's caution regarding the interpretation of Streptococcus and *Cutibacterium*. As we demonstrated in our baseline dilution series (Figure 7), these two genera are ubiquitous components of background environmental noise under low-biomass conditions. Therefore, to ensure methodological consistency and prevent the overinterpretation of experimental artifacts, we established a strict biomass-dependent interpretive rule in our revised manuscript: In low-biomass samples (Cq $\geq$ 25.5), the detection of Streptococcus and *Cutibacterium* is conservatively treated as environmental/reagent contamination (as summarized in Supplementary Table S3). Conversely, in high-biomass samples (Cq $\leq$ 25.4) where the target DNA density is high and *decontam* filters are applied, these signals cannot be purely dismissed as noise. In Case 2, once the overwhelming *Streptophyta* noise was mathematically removed in Step 2, the underlying polymicrobial profile of this high-biomass specimen was unmasked. Because this high-biomass sample satisfies our established quantitative cutoff, these surviving taxa are highly likely to reflect true, low-grade subclinical colonization or a polymicrobial biofilm in vivo (which may include commensal or skin/oral-derived flora) rather than a simple low-biomass artifact. We have carefully refined our taxonomic discussion in the text to reflect this biomass-dependent distinction,

avoiding any definitive or speculative overcharacterization of the exact bacterial origin while maintaining strict scientific rigor. We believe that these quantitative comparative profiles, clarified filtering protocols, and refined interpretive boundaries successfully resolve the reviewer's concerns and ensure the logical consistency of our metagenomic pipeline.

Comment3

Clinical performance reporting: Provide sensitivity/specificity/ PPV/NPV for qPCR, NGS, and the combined strategy using your infection definition; account for multiple samples per patient.

Response3:

We sincerely thank the reviewer for this pivotal statistical recommendation. We agree that in clinical cohort studies evaluating multiple anatomical or intraoperative specimens per individual patient, accounting for the interdependence of clustered samples is mandatory to ensure the statistical validity of sensitivity, specificity, positive predictive value (PPV), and negative predictive value (NPV).

To address the reviewer's request completely, we have rigorously accounted for the presence of multiple samples per patient across both our large qPCR screening cohort (N = 56 patients; 276 total samples) and our targeted NGS cohort (N = 15 patients; 83 total samples), utilizing stratified patient-level consensus criteria:

1. In the qPCR Screening Cohort (N = 56 patients, 276 samples; Tables 5 and 6):

To resolve the statistical dependency of multiple peri-implant tissue and joint aspirate cultures obtained from the same individual, we shifted our unit of analysis from individual specimens to patient-level diagnostic thresholds. As detailed in Table 7, we evaluated performance based on defined patient-level criteria (≥ 1 positive culture versus ≥ 2 positive cultures). Furthermore, in Table 8, we explicitly demonstrated the clinical performance of combined strategies based on these consensus definitions. Defining positivity via a strict patient-level consensus—requiring either ≥ 2 positive cultures or 1 positive culture concurrently paired with a positive qPCR result (Cq ≤ 25.4)—effectively neutralized the clustering effect of multiple samples. This strategy achieved a highly dependable, balanced profile with a 100% specificity of 100% (95% CI: 88.0%–100%) and a solid sensitivity of 80.0% (95% CI: 55.7%–93.3%).

2. In the Targeted NGS Cohort (N = 15 patients, 83 samples; Table 7, 8 and 9):

Consistent with the qPCR methodology, we applied identical patient-level clustering adjustments to our sequencing cohort. As compiled in the newly finalized Table 9, standalone sonicate fluid qPCR demonstrated a sensitivity of 100% but was prone to low-biomass background noise when evaluated on an unadjusted single-specimen basis (standalone specificity of 72.7%). However, when integrating the qPCR threshold with downstream NGS processing via the *decontam* package using the strict patient-level consensus criterion (≥ 1 positive PCR with NGS), our workflow successfully eradicated all low-biomass false-positive signals among the 11 non-infected controls, achieving a 100% specificity of 100% (95% CI: 68%–100%) and a PPV of 100% (95% CI: 31%–100%).

All mathematical metrics, including exact denominators, numerators, and 95% confidence intervals (calculated using Clinical Calculator 1 to ensure statistical precision), have been fully updated and are detailed across Tables 7, 8, and 9 in the revised manuscript. We believe this comprehensive, multi-cohort reporting directly provides the quantitative context requested and completely resolves the methodological query.

Comment 4:

Bioinformatics choices: Justify >3% "significant genus" cutoff, 10k rarefaction, OTU97%, and Greengenes use; discuss how these choices affect pathogen detection.

Response:

We sincerely thank the reviewer for this critical methodological query regarding our bioinformatic parameters. We agree that cross-validating and justifying these analytical choices with established literature is essential to demonstrate the fidelity and reproducible nature of our pathogen detection pipeline.

In accordance with your recommendation, we have expanded both the Methods and Discussion sections of the revised manuscript to explicitly articulate the scientific rationale for each choice, citing key references from our literature list to demonstrate their universal validity:

1. Rationale for the ≥ 3% "Significant Genus" Cutoff:
    - Justification: In low-biomass clinical specimens, such as orthopedic sonication fluids, high-throughput sequencing is highly susceptible to generating trace-level false-positive reads arising from cross-contamination or ambient dust during laboratory handling. Establishing ≥ 3% relative abundance threshold functions as a rigorous filtering step to isolate true, clinically relevant dominant pathogens from this minor experimental noise. This conservative strategy directly aligns with previously validated diagnostic microbiome frameworks by Tarabichi et al. [6] and Street et al. [5], which utilized similar abundance thresholds to control for background contamination in PJI and orthopedic-device-related infection cohorts.
    - Impact on Pathogen Detection: While this conservative threshold theoretically introduces a minor risk of missing extremely low-abundance organisms, it drastically enhances diagnostic specificity and positive predictive value, preventing the overinterpretation of technical noise as clinical infections, a critical concern raised in historical orthopedic NGS evaluations [4–7]. In clinical practice, genuine PJI pathogens typically proliferate and dominate the local joint environment, consistently exceeding 3% abundance upon data normalization.
2. Rationale for the 10,000 (10k) Rarefaction Depth:
    - Justification: Rarefaction to 10,000 reads per sample was implemented to standardize sequencing depth variations across independent samples, ensuring uniform statistical comparisons of relative abundance. This 10k read threshold is a widely accepted and highly validated benchmark in clinical 16S rRNA gene sequencing studies, including the landmark metagenomic sonication fluid study by Street et al. [5], providing a rigorous balance between sub-sampling normalization and data retention. Our baseline validation plots confirmed that the alpha-diversity rarefaction curves achieved a clear plateau at approximately 5,000 to 7,000 reads for all clinical specimens.
    - Impact on Pathogen Detection: Standardizing at 10k reads ensures sufficient sequencing coverage to capture the entire microbial diversity of the samples without introducing artificial variance from over-sequenced or under-sequenced datasets.
3. Rationale for the 97% Sequence Identity Threshold for OTU Clustering:
    - Justification: The 97% sequence identity threshold represents the classic gold standard and historically validated benchmark for genus-to-species level operational taxonomic unit (OTU) clustering in 16S rRNA gene amplicon sequencing, based on the foundational taxonomic criteria established by Stackebrandt and Goebel (1994). It remains a standard paradigm for pathogen detection in periprosthetic joint infections.
    - Impact on Pathogen Detection: Utilizing a 97% similarity threshold balances taxonomic resolution with technical error tolerance. It effectively clusters minor sequencing artifacts caused by polymerase errors into single, high-confidence taxonomic groups, preventing the artificial inflation of rare taxa while ensuring reliable identification of core PJI pathogens at the genus level (e.g., *Staphylococcus*, *Streptococcus*).
4. Rationale for Utilizing the Greengenes Database:
    - Justification: The Greengenes database (v13_8; DeSantis et al., 2006) was selected because it is deeply integrated into standard, reproducible workflows (such as QIIME) and remains one of the most comprehensively validated references for annotating skin, oral, and environmental commensal flora—which represent the primary sources of both real PJI pathogens and potential background contaminants evaluated in contemporary orthopedic NGS research.
    - Impact on Pathogen Detection: While newer databases exist, Greengenes ensures strong backward compatibility and direct comparability of our data with a massive body of historical PJI-microbiome literature. As explicitly noted in our text and Table 8 footnotes, any database-specific nomenclature variances (such as the legacy annotation of *Propionibacterium* instead of *Cutibacterium*) were thoroughly cross-referenced to prevent any clinical ambiguity.

We believe that these explicit, literature-backed methodological justifications reinforce the technical

robustness of our diagnostic framework.

Minor points
Comment 5:
Comments on Details: (e.g., minor issues regarding terminology alignment like *Propionibacterium* versus *Cutibacterium*, clarity on cohort aggregation, and the addition of a conceptual flowchart to visualize the screening-to-sequencing workflow).
Response 5:
We sincerely thank the reviewer for these highly constructive editorial suggestions regarding the technical details and structural clarity of our manuscript. In accordance with your recommendations, we have thoroughly updated our data representation and revised the introductory visual materials:
1. Standardization of Microbiological Terminology:
We have meticulously cross-referenced the entire manuscript, including all figures and tables, to standardize and harmonize our taxonomic terminology. Specifically, any legacy annotations generated by the Greengenes database (e.g., *Propionibacterium*) have been explicitly aligned and synthesized with the current official nomenclature (*Cutibacterium*) utilized throughout the main text, thereby eliminating any potential cross-referencing ambiguity for the reader.
2. Clarification of Cohort Aggregation and Sample Flow:
To ensure absolute transparency regarding our sample data aggregation, we have thoroughly updated our experimental overview. We have integrated a concise, high-visibility clinical flowchart to ensure that the strict progression from initial patient screening to ultimate high-throughput downstream sequencing is immediately comprehensible within the first two introductory figures:

- Figure 1 (Study Flowchart): Revised to systematically outline the exact quantitative metrics at each phase, explicitly detailing the total number of enrolled patients (N = 56 for qPCR screening; n = 15 for targeted NGS profiling), the exact number of distinct clinical specimens collected per cohort (276 pre/intraoperative tissue and fluid samples vs. 83 sonicated fluid samples), and the strict diagnostic stratification criteria applied.
- Figure 2 (Analytical Sequencing Workflow): Enhanced to document the baseline bioinformatic filtering framework, clarifying the total number of raw sequences analyzed, the exact negative and positive reference boundaries, and the step-by-step reduction of environmental artifacts.

We believe that these refined detail alignments and newly integrated visualizations have drastically enhanced the readability and technical robustness of our methodological framework.

Reviewer #2 (Comments to the Author):
Comment:
This manuscript addresses an important and increasingly relevant topic in the diagnosis of periprosthetic joint infection (PJI): the integration of quantitative PCR (qPCR) and 16S rRNA amplicon sequencing using identical primers, with a focus on bacterial biomass and contamination in low biomass samples. The study design is thoughtful, the use of multiple controls is commendable, and the clinical relevance is high. However, several key issues related to structure, statistical rigor, and interpretation must be addressed.

RESPONSE TO REVIEWER #2's GENERAL COMMENTS
We would like to express our sincere gratitude to Reviewer #2 for the time, effort, and highly encouraging feedback dedicated to our manuscript. We are deeply grateful for your positive evaluation of our study design, our implementation of multiple experimental controls (which you noted as commendable), and the high clinical relevance of integrating qPCR with 16S rRNA amplicon sequencing to resolve the complex diagnosis of low-biomass implant infections. We agree that enhancing the structural clarity, statistical rigor, and taxonomic interpretation of our data is essential to maximize the validity and clinical impact of our findings, particularly given the subclinical nature of low-biomass prosthetic joint infections (PJIs) and the

persistent challenge of distinguishing infectious from non-infectious implant loosening. In accordance with your expert recommendations, we have performed a comprehensive revision of our manuscript and have systematically implemented the following structural and statistical updates:

1. Enhancement of Statistical Rigor: To address the critical issue of statistical independence when analyzing multiple intraoperative tissue and fluid specimens per individual patient, we have formalized our clinical performance reporting using stratified patient-level consensus criteria. Complete sensitivity, specificity, PPV, and NPV metrics (including exact 95% confidence intervals) have been systematically updated across our cohorts (now detailed in Tables 7, 8, and Table 9).
2. Refinement of Taxonomic Interpretation and Contamination Handling: We have established a strict, biomass-dependent interpretive framework to distinguish true biological signals from environmental artifacts under low-biomass conditions. This prevents any overinterpretation of common background contaminants based on relative abundances, allowing for a safer and more nuanced differentiation of subclinical colonization from aseptic loosening.
3. Structural and Terminological Standardization: We have streamlined the structural narrative of both the Results and Discussion sections, including the integration of a concise workflow flowchart (Figure 1) and the standardization of all microbiological nomenclature (cross-referencing legacy annotations to current taxonomy) to optimize readability and formatting consistency. Below is our point-by-point response to the specific major and minor issues raised, detailing the exact modifications made to the revised manuscript. We believe these extensive updates successfully resolve all your concerns and significantly enhance the scientific rigor of our work.

Major Comments

Comment1:

1. Clarity and focus of the Introduction

The Introduction is informative but overly long and diffuse. Multiple themes-low-biomass challenges, contamination, primer bias, limitations of previous orthopedic NGS studies, and the study's aims-are interwoven in a way that obscures the core rationale. Please restructure this section and present a concise paragraph outlining the specific aims of the study early in the Introduction

Response1: We sincerely thank the reviewer for this excellent and highly constructive structural recommendation. We agree that the original Introduction interwoven too many sub-themes simultaneously, which obscured the core clinical rationale and delayed the presentation of our research objectives. In accordance with your expert guidance, we have thoroughly restructured and streamlined the Introduction to enhance its clarity and narrative focus.

We have compiled a concise, dedicated paragraph clearly outlining the specific aims of this study and positioned it immediately following this established rationale, ensuring that our research objectives are explicitly communicated early in the manuscript. The newly refined paragraph outlining the study's specific aims has been added to the Introduction as follows: "To address these critical diagnostic limitations in low-biomass environments, the primary objective of this study was to establish and validate a pragmatic, highly reproducible molecular diagnostic workflow for PJI by utilizing identical universal primers for both broad-range 16S rRNA gene quantitative PCR (qPCR) screening and NGS. Specifically, we aimed to: (i) determine a statistically optimized, parameter-driven qPCR Cq threshold capable of identifying the precise tipping point where biological signals become dominated by contamination; (ii) evaluate the diagnostic performance of this integrated quantitative pipeline against conventional microbiological cultures across a large clinical cohort; and (iii) demonstrate the clinical utility of this molecular framework in uncovering occult subclinical pathogens and preventing the underdiagnosis or misclassification of low-biomass implant infections." We believe that this structural reorganization has significantly improved the readability, scientific focus, and impact of our Introduction.

Comment2:

2. Statistical analysis

Although the experimental work is robust, the manuscript lacks statistical evaluations. For example, no

statistical comparison between high- and low-biomass groups, no confidence intervals for determining the Cq cutoff, no evaluation of contamination taxa using available computational or statistical tools. Please include at least basic statistical analyses (e.g., Mann-Whitney U tests, Wilcoxon signed-rank tests, or measures of variability for control samples).

Response2:
We sincerely thank the reviewer for this pivotal statistical recommendation. We agree that reinforcing our robust experimental work with rigorous statistical evaluations and explicit variability metrics is essential to validate our clinical conclusions. In accordance with your expert guidance, we have thoroughly integrated a comprehensive statistical framework across our datasets, moving away from descriptive summaries to parameter-driven mathematical evaluations. Specifically, we have updated the Methods, Results, and corresponding Tables/Figures to detail these analyses as follows:
1. Statistical Comparison between High- and Low-Biomass Groups: To objectively characterize the bacterial population behavior across biomass strata, we systematically calculated the correlation coefficients and corresponding statistical significance (P values) between qPCR Cq values and the relative abundance of predominant taxa. As now detailed in the newly finalized “Supplementary Table S3”, we applied the Mann–Whitney U test and Pearson’s chi-squared test to evaluate variations between the high-biomass ($Cq \leq 25.4$) and low-biomass ($Cq \geq 25.5$) cohorts. Moderate correlations were observed between Cq values and the relative abundances of *Sediminibacterium*, MLE1-12, and *Sinobacteraceae* among the top ten most abundant bacterial taxa identified by NGS in the implant sonicated fluid samples. A weak correlation was also observed for *Sphingomonadaceae* (see Supplementary Table S3). Consistent with our previous analyses demonstrating a positive correlation between Cq values and the relative abundance of specific background bacteria, these taxa were classified as likely contaminants and systematically removed using *decontam* (Supplementary Table S3).
2. Integration of Confidence Intervals for Diagnostic Thresholds: To establish the statistical precision and reliability of our NGS-derived Cq cutoff, we calculated exact 95% confidence intervals (95% CI) for sensitivity, specificity, positive predictive value (PPV), and negative predictive value (NPV) using Clinical Calculator 1 (18, 29). As presented in the revised “Table 5, 6, and 9”; (Table 5. Comparison of microbiologic test), (Table 6. Comparison of diagnostic performance among combined culture), and the newly added Table 9 (clinical performance report for the 15-patient NGS cohort), these rigorous interval metrics mathematical validate the diagnostic stability and reproducibility of our screening protocol.
3. Evaluation of Contamination Taxa via Computational/Statistical Tools: Rather than relying on visual inspection alone, we employed the prevalence-based statistical framework within the R package *decontam* to mathematically isolate true biological signals from environmental artifacts. To ensure the robustness of this computational tool, we conducted a prevalence threshold sensitivity analysis (evaluating a range from 0.1 to 0.5). As illustrated in the newly compiled “Supplementary Figure S1”, statistical measures of variability and taxonomic retention confirmed that a threshold parameter of 0.3 provided the optimal mathematical consensus to eradicate background noise while successfully preserving true positive control signals. Furthermore, we have explicitly documented the specific statistical modalities utilized—including the Mann–Whitney U test, Fisher’s exact test, and Pearson’s chi-squared test—within the Methods section and in the detailed footnotes of Tables 1 and Table 3 to ensure total methodological transparency. We believe these extensive mathematical supplementations fully address the reviewer's concerns and have significantly enhanced the scientific rigor of our manuscript.

Comment3:
3. Justification and generalizability of the $Cq = 25.5$ cutoff; ($Cq \leq 25.4$).
The cutoff is experimentally derived using E. coli, which is reasonable, but the manuscript does not discuss its applicability to other taxa or primer bias associated with the V1-V2 region. Please elaborate on the limitations of using a single organism to establish a universal threshold

Response3:

We sincerely thank the reviewer for this exceptionally sharp and critical methodological insight. We agree that using a single organism Escherichia coli to establish an analytical baseline, alongside potential primer biases associated with targeting the V1–V2 hypervariable region of the 16S rRNA gene, presents inherent limitations regarding universal generalizability that must be thoroughly articulated.

In accordance with your expert recommendation, we have significantly expanded the "Limitations of the study" section in the revised manuscript to explicitly address these physiological and technological constraints, presenting a balanced discussion on why this model was chosen and how it affects pathogen detection:

1. Scientific Rationale and Safety Margin of the E. coli Model: As we have now detailed in our revised text, adopting *E. coli* (a Gram-negative organism) serves as a deliberate "conservative threshold design." Broad-range 16S rRNA universal primers targeting the V1–V2 region can exhibit lower thermodynamic amplification efficiencies for certain Gram-negative lineages compared to the robust, highly efficient amplification typically observed for Gram-positive pathogens (such as *Staphylococcus* species, which are responsible for the vast majority of PJIs). By establishing our quality-control cutoff based on the higher limit of detection required for this more difficult-to-amplify Gram-negative model, our workflow inherently guarantees a wide safety margin that reliably captures Gram-positive PJI pathogens at equivalent or even lower biological densities.

2. Elaboration on Primer Bias and V1–V2 Region Constraints: We fully acknowledge that single-region amplicon sequencing cannot achieve a perfect taxonomic resolution across all bacterial species due to inherent hypervariable region sequence variations. To transparently communicate this limitation to the scientific community, we have thoroughly updated and expanded the first and second paragraphs of the "Limitations of the study" section, explicitly highlighting the technical boundary of our short-read approach and emphasizing the necessity of cross-validating these findings against alternative sequencing platforms and variable genetic regions. The exact expanded text has been integrated into the "Limitations of the study" section as follows:

First, the clinical quantification cycle (Cq) values and established thresholds reported herein are inherently subject to inter-facility and inter-instrument variations, meaning they cannot be directly generalized without local calibration. The precise reliability and reproducibility of these absolute Cq metrics must be independently validated against characterized bacterial loads at individual testing facilities.

Second, our total sample size was relatively limited... Furthermore, extensive validation of identical clinical specimens under diverse experimental conditions remains necessary. This includes evaluations across different laboratories and on alternative sequencing platforms—such as short-read technologies like Illumina or long-read platforms like Oxford Nanopore vs. the Ion Torrent platform utilized here. Additionally, future studies should investigate targeting different hypervariable regions of the 16S rRNA gene (or the full-length gene via long-read sequencing), as well as utilizing alternative master mixes and DNA extraction reagents. Comprehensive monitoring and multi-center cross-validation are required to definitively ensure the universal robustness and reproducibility of our proposed diagnostic framework." We believe that this literature-backed, balanced discussion on our bioinformatic constraints and primer considerations successfully resolves the reviewer's concerns and reinforces the technical transparency of our study.

Comment4:

4. Interpretation of low-biomass findings

Some statements-particularly those regarding *Streptophyta* and oral bacteria potentially contributing to implant loosening-are too strong given the possibility of contamination and the constraints of 16S methods.

Response4:

We sincerely thank the reviewer for this critical and highly constructive pedagogical insight. We agree that our original statements lacked sufficient caution regarding the inherent constraints of low-biomass 16S rRNA sequencing and the potential distortion caused by environmental or chloroplast-derived artifacts. In accordance with your expert recommendation, we have thoroughly revised and refined our taxonomic interpretation within the Discussion section. We have systematically toned down any definitive or

speculative assertions, transitioning from a causal statement to a parameter-driven, suggestive clinical hypothesis. Specifically, we implemented the following two major updates:

1. Clarification of *Streptophyta* as a Non-Clinical Artifact: We have explicitly clarified in the revised text that the predominance of *Streptophyta* (accounting for 90% of raw sequences in Case 2) represents non-clinical, plant-derived chloroplast noise. To prevent this noise from skewing our clinical inference, we utilized a rigorous organellar filtering framework in our bioinformatic pipeline (Methods section), which successfully eradicated the *Streptophyta* lineage prior to downstream interpretation.

2. Implementation of a Biomass-Dependent Interpretive Framework for Oral Flora Inference: To maintain methodological consistency and prevent the overinterpretation of experimental noise, we established a strict biomass-stratified ruleset. Under low-biomass conditions ($Cq \geq 25.5$), ubiquitous genera like Streptococcus are conservatively treated as ambient contamination (Supplementary Table S3). Conversely, Case 2 satisfied our high-biomass quantitative threshold ($Cq \leq 25.4$). Once the overwhelming chloroplast noise was filtered out, the underlying high-confidence biological profile was unmasked, revealing a stable polymicrobial signature dominated by *Streptococcus* (20%), *Cutibacterium* (17%), *Anaerococcus* (14%), *Paracoccus* (11%), and *Staphylococcus* (7%) (Supplementary Table S5). Because these specific *Streptococcus* lineages are characteristic of predominant oral microbiota, we have carefully refined our discussion. Rather than asserting a direct mechanical cause, we now propose that these high-confidence molecular signals imply that the subclinical colonization of oral-derived commensal flora potentially contributes to the pathogenesis of delayed implant loosening through a low-grade biofilm framework. The corresponding paragraph in the Discussion section (under "NGS analysis of qPCR-positive cases with occult infections") has been modified as follows to ensure strict academic rigor: "Importantly, interpreting *Streptococcus* and *Cutibacterium* requires caution because both genera are common environmental contaminants under low-biomass conditions ($Cq \geq 25.5$), as demonstrated in our baseline validation (Figure 7, Supplementary Table S3). To maintain strict logical consistency, we implemented a biomass-dependent interpretive framework. While these taxa are conservatively treated as contamination in low-biomass samples, they reflect true biological signals in high-biomass samples ($Cq \leq 25.4$). In Case 2, once the overwhelming chloroplast-derived *Streptophyta* noise was completely removed via organellar filtering, the underlying high-biomass polymicrobial profile was successfully unmasked. Because this specimen satisfied our validated quantitative cutoff, the persistence of *Streptococcus* (20%), *Cutibacterium* (17%), *Anaerococcus* (14%), *Paracoccus* (11%), and *Staphylococcus* (7%) (Supplementary Table S5) cannot be dismissed as a technical artifact. Given that these identified *Streptococcus* lineages are characteristic of predominant human oral microbiota, these molecular findings imply that the subclinical colonization of oral/skin-derived commensal flora potentially contributes to the pathogenesis of delayed implant loosening through a low-grade, polymicrobial included oral flora bacteria and skin flora bacteria forming biofilm framework, rather than representing an aseptic mechanical failure." We believe these extensive modifications have successfully resolved the overinterpretation, perfectly aligning our clinical narrative with the strict technical boundaries of molecular diagnostics.

Comment5:

5. Figures

The manuscript contains numerous figures, some of which are highly detailed. This makes it difficult for readers to identify the main findings. Please consider moving detailed or case-specific figures (e.g., full NGS histograms for all samples) to the Supplementary Materials.

Response5:

Detailed figures and tables, as well as those pertaining to specific cases, have been moved to the supplementary materials. Additionally, while the paper has come to include several more figures and tables as a result of additional verification based on reviewers' feedback, we have included only the necessary ones in the main manuscript and placed all supplementary data in the supplementary materials.

Re: Spectrum04128-25R1 (The Diagnostic Potential of Combined Quantitative Polymerase Chain Reaction and Next-Generation Sequencing Using the Same Primers for Periprosthetic Joint Infection)

Dear Dr. Narumi Ueda:

Your manuscript has been accepted, and I am forwarding it to the ASM production staff for publication. Your paper will first be checked to make sure all elements meet the technical requirements. ASM staff will contact you if anything needs to be revised before copyediting and production can begin. Otherwise, you will be notified when your proofs are ready to be viewed.

Data Availability: ASM policy requires that data be available to the public upon online posting of the article, so please verify all links to sequence records, if present, and make sure that each number retrieves the full record of the data. If a new accession number is not linked or a link is broken, provide production staff with the correct URL for the record. If the accession numbers for new data are not publicly accessible before the expected online posting of the article, publication may be delayed; please contact ASM production staff immediately with the expected release date.

Publication Fees: For information on publication fees and which article types have charges, please visit our website. We have partnered with Copyright Clearance Center (CCC) to collect author charges. If fees apply to your paper, you will receive a message from no-reply@copyright.com with further instructions. For questions related to paying charges through RightsLink, please contact CCC at ASM_Support@copyright.com or toll free at +1-877-622-5543. CCC makes every attempt to respond to all emails within 24 hours.

ASM Membership: Corresponding authors may join or renew ASM membership to obtain discounts on publication fees. Need to upgrade your membership level? Please contact Customer Service at Service@asmusa.org.

Embargo Policy: A press release may be issued as soon as the manuscript is posted on the Spectrum Latest Articles webpage. The corresponding author will receive an email with the subject line "ASM Journals Author Services Notification" when the article is available online.

The ASM Journals program strives for constant improvement in our submission and publication process. Please tell us how we can improve your experience by taking this quick Author Survey.

Thank you for submitting your paper to Spectrum.

Sincerely,
Takeaki Wajima
Editor
Microbiology Spectrum

Reviewer #1 (Comments for the Author):

Authors have addressed all the concerns previouly raised

Reviewer #2 (Comments for the Author):

The authors have adequately addressed all my comments, and I have no further concerns.
