# Supplementary material for: The diagnostic potential of combined quantitative polymerase chain reaction and next-generation sequencing using the same primers for periprosthetic joint infection
Source: Microbiol Spectr. 2026 Jul 28;14(9):e04128-25. doi: 10.1128/spectrum.04128-25 (PMC13532281; doi:10.1128/spectrum.04128-25)
Supplement: Supplementary file 2 [file spectrum.04128-25-s0001.pdf]

Supplementary Figure S1.

Supplementary Figure S1

Decontam cut off	The number of taxonomies that were originally included in MOCK but were accidentally excluded	Taxonomies excluded counts removal rate (%)	Taxonomy numbers excluded from the control group removal rate (%)	Taxonomy numbers (negative control) of removal rate (%)
0.1	0	3054/51217 (6%)	14/90 (16%)	10/30 (33%)
0.2	0	11906/51217 (23%)	27/90 (30%)	20/30 (67%)
0.3	0	17146/51217 (33%)	35/90 (39%)	26/30 (87%)
0.4	2	19585/51217 (38%)	40/90 (44%)	26/30 (87%)
0.5	4	20182/51217 (39%)	46/90 (51%)	27/30 (90%)

Supplementary Figure S1 Legend

Evaluation of prevalence thresholds in the *decontam* algorithm using the mock community control. The graph demonstrates the impact of varying prevalence thresholds (0.1, 0.2, 0.3, 0.4, and 0.5) on the classification of operational taxonomic units (OTUs). Expected mock-community bacterial signals are successfully retained at a threshold of 0.3. However, at thresholds of 0.4 and above, these true biological signals begin to be misclassified as candidate contaminants. Based on these findings, 0.3 was determined as the optimal and strictest threshold to maximize decontamination efficiency while preserving the expected mock-community profile.

Supplementary Figure S2.

Supplementary Figure S2

Result; Next-generation sequencing

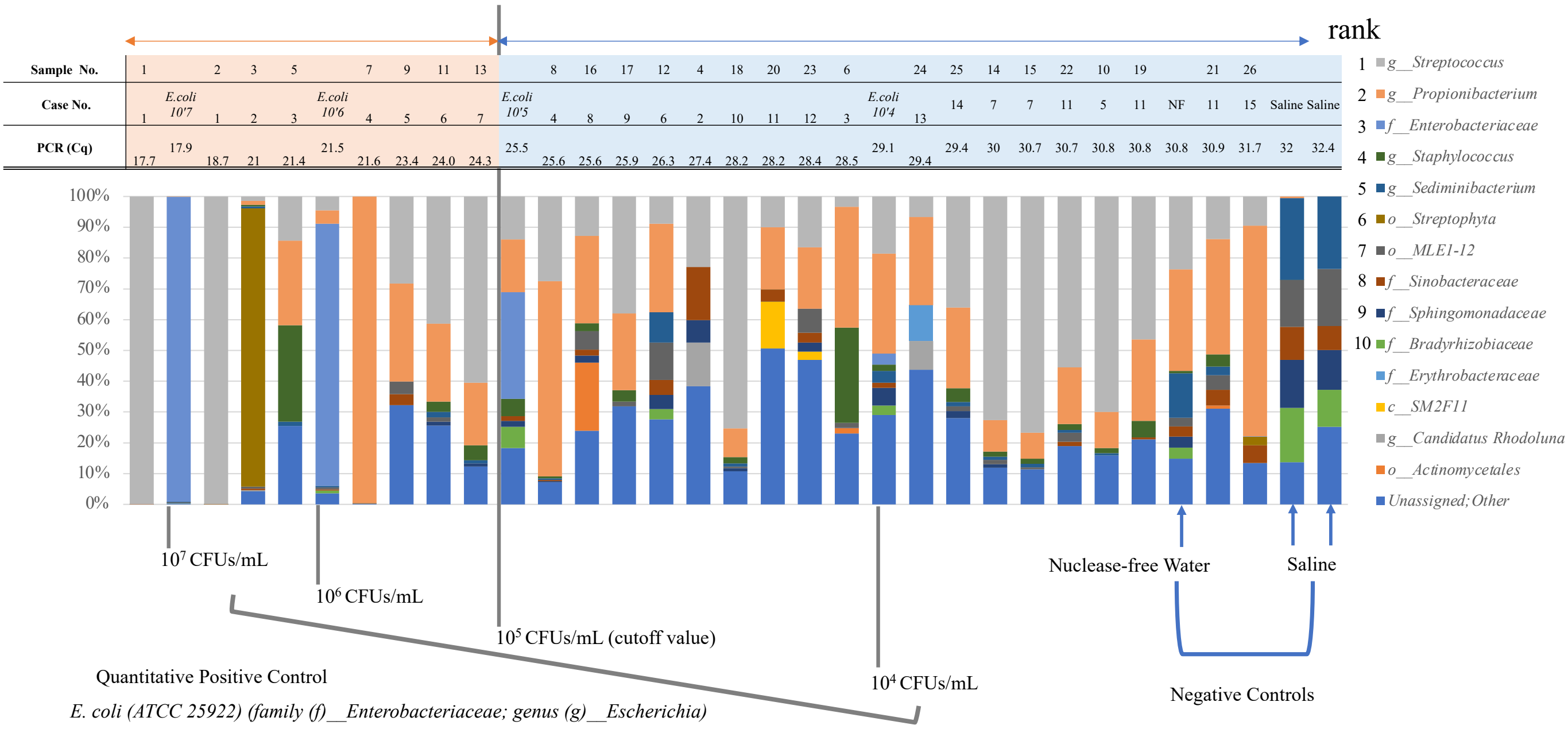

Supplementary Figure S2. Overview of next-generation sequencing (NGS) microbial profiles and corresponding qPCR Cq values across clinical and control samples.

The stacked bar chart illustrates the relative taxonomic abundance of bacterial taxa across all analyzed specimens, quantitative positive controls, and negative controls. Samples on the horizontal axis are ordered by bacterial DNA quantity based on qPCR Cq values, which are stratified into high-biomass and low-biomass regions by the established threshold (Cq = 25.5, corresponding to 10^5 CFU/mL. Black Lines at the bottom indicate the quantitative positive controls (*Escherichia coli* ATCC 25922) ranging from 10^7 to 10^4 CFU/mL. Negative controls include nuclease-free water and sterile saline, indicated by the blue bracket. The right-hand panel lists the predominant bacterial taxa ranked by their overall relative abundance. Note that *g__Propionibacterium* annotated by the Greengenes database corresponds to *Cutibacterium* referred to in the main text.

Supplementary Figure S3.

Supplementary Figure S3

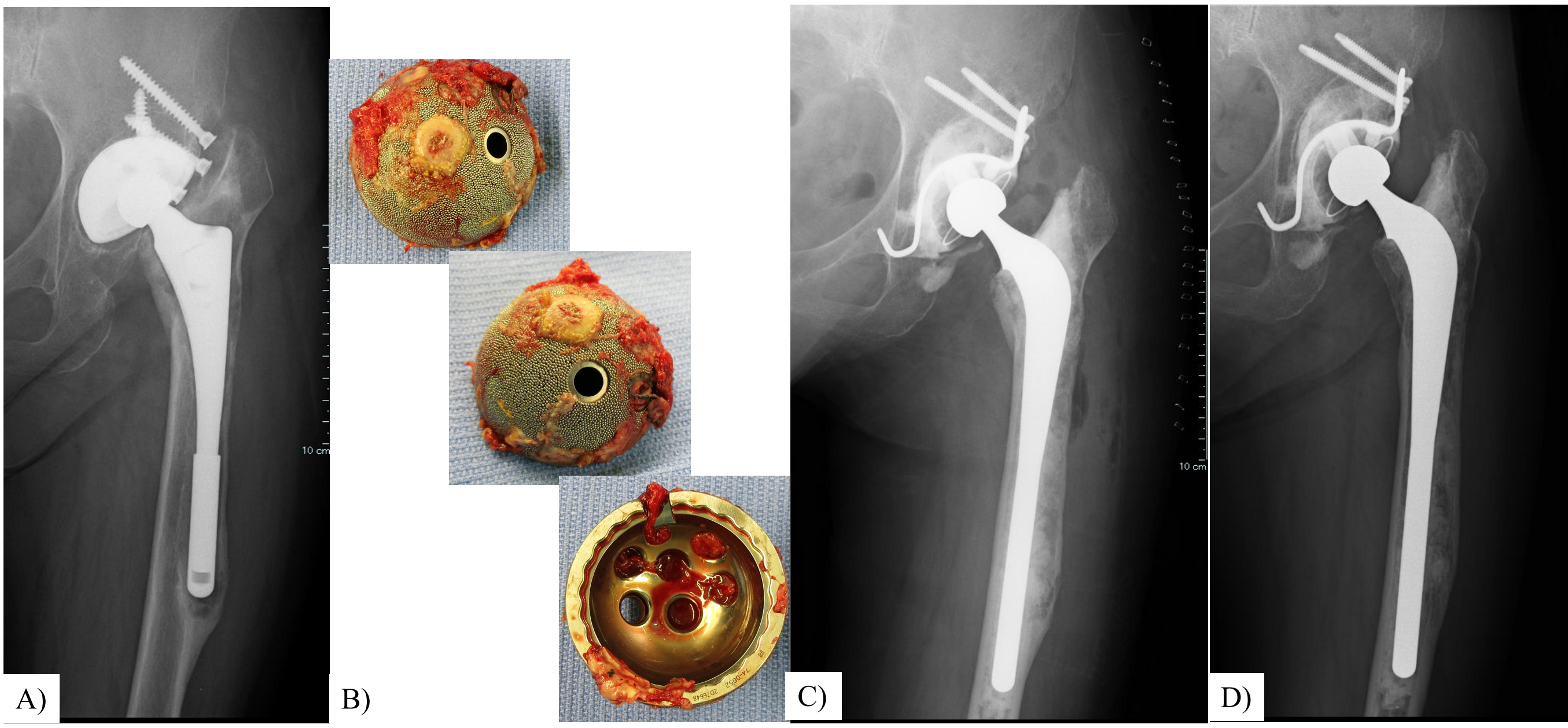

Supplementary Figure S3. Clinical course and radiographic evaluation of Case 2.
A 73-year-old female presented with unspecific left hip pain and apparent leg length discrepancy 20 years after primary uncemented total hip arthroplasty (THA). Inflammatory biomarkers were only slightly elevated (C-reactive protein, 3.0 mg/L; D-dimer, 1.0 μ g/mL). **A)** Preoperative radiograph demonstrating aseptic-like loosening of both the acetabular cup and the femoral stem, accompanied by a marked periacetabular osseous defect and proximal femoral osteolysis. A one-stage revision arthroplasty using cemented components was performed under suspicion of septic loosening. **B)** Gross pathology of the retrieved acetabular component. Although conventional cultures from implant sonication fluid remained negative, the specimen yielded a positive qPCR result and next-generation sequencing (NGS) analysis identified multiple bacterial species, including *Staphylococcus*, may have colonized the implant surface. **C)** Postoperative radiograph showing reconstruction of the bone defects filled with antibiotic-impregnated bone allografts. Postoperative management included 4 weeks of intravenous antibiotic therapy followed by oral antibiotics. **D)** Radiograph obtained 4 years postoperatively. The patient remains completely pain-free with no clinical signs of recurrent infection. Complete remodeling of the bone allograft was achieved.

Supplementary Figure S4.

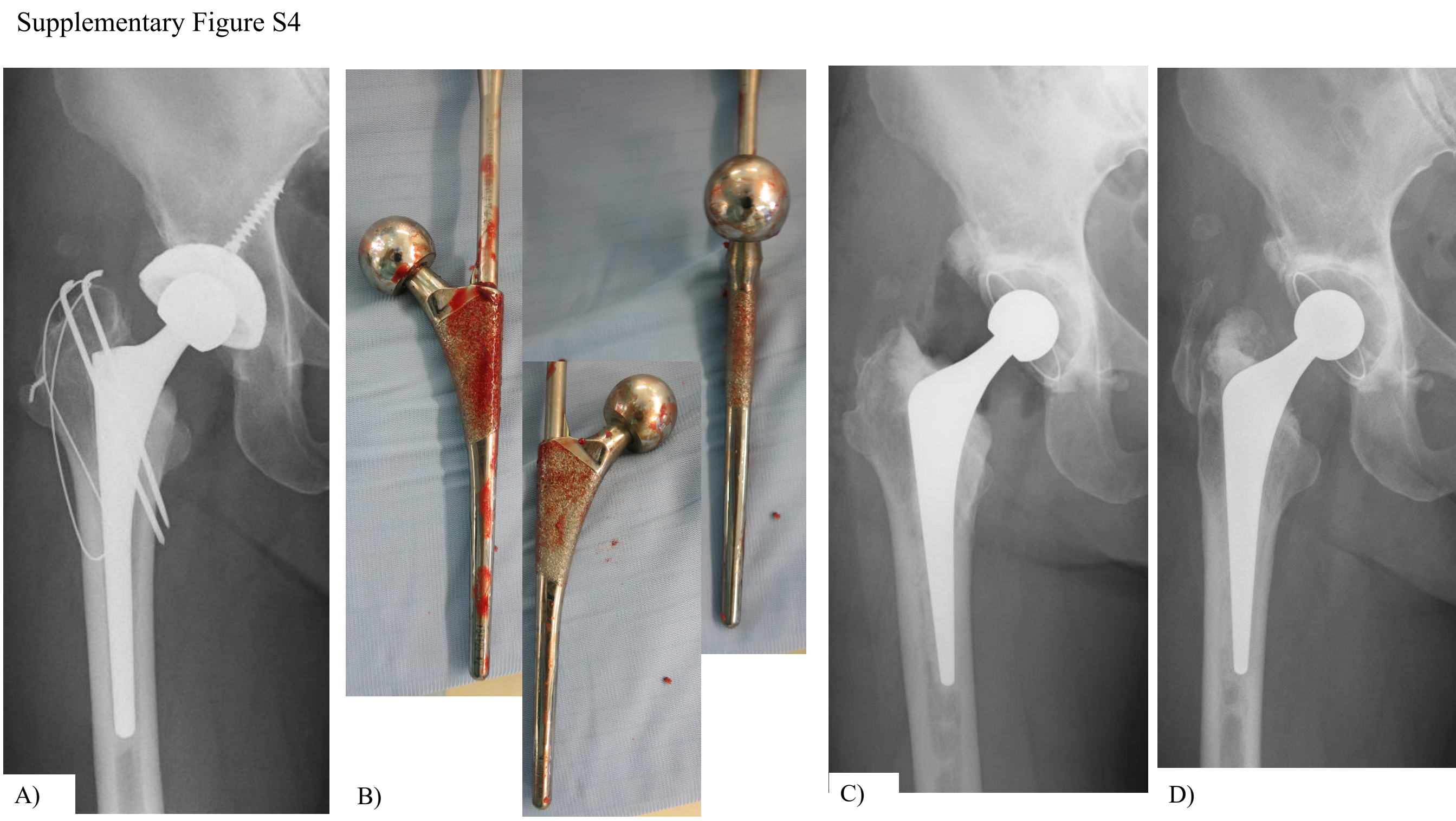

Supplementary Figure S4. Clinical course and radiographic evaluation of Case 5.
A 58-year-old female presented with progressive, nonspecific pain radiating from the knee to the hip two years after undergoing primary cementless total hip arthroplasty (THA). Inflammatory biomarkers were within normal ranges; however, her pain gradually worsened, severely limiting ambulation and requiring a walking cane. A one-stage revision THA was performed under clinical suspicion of stem loosening and periprosthetic joint infection (PJI). A) Preoperative radiograph demonstrating features consistent with implant loosening. B) Intraoperative photographs of the retrieved cementless femoral component covered in blood and tissue debris. The stem was highly loose and easily extracted under sterile conditions. Combined molecular analysis of the residual implant fluid subsequently yielded a positive qPCR result and identified pathogen candidates via next-generation sequencing (NGS). C) Postoperative radiograph showing one-stage exchange using cemented components. The acetabular and femoral bone defects were successfully reconstructed using antibiotic-impregnated bone grafts. D) Radiograph obtained 4 years postoperatively. The patient remains completely pain-free with no clinical signs of recurrent infection. Favorable remodeling and incorporation of the bone allograft were achieved.

Supplementary Figure S5.

Supplementary Figure S5

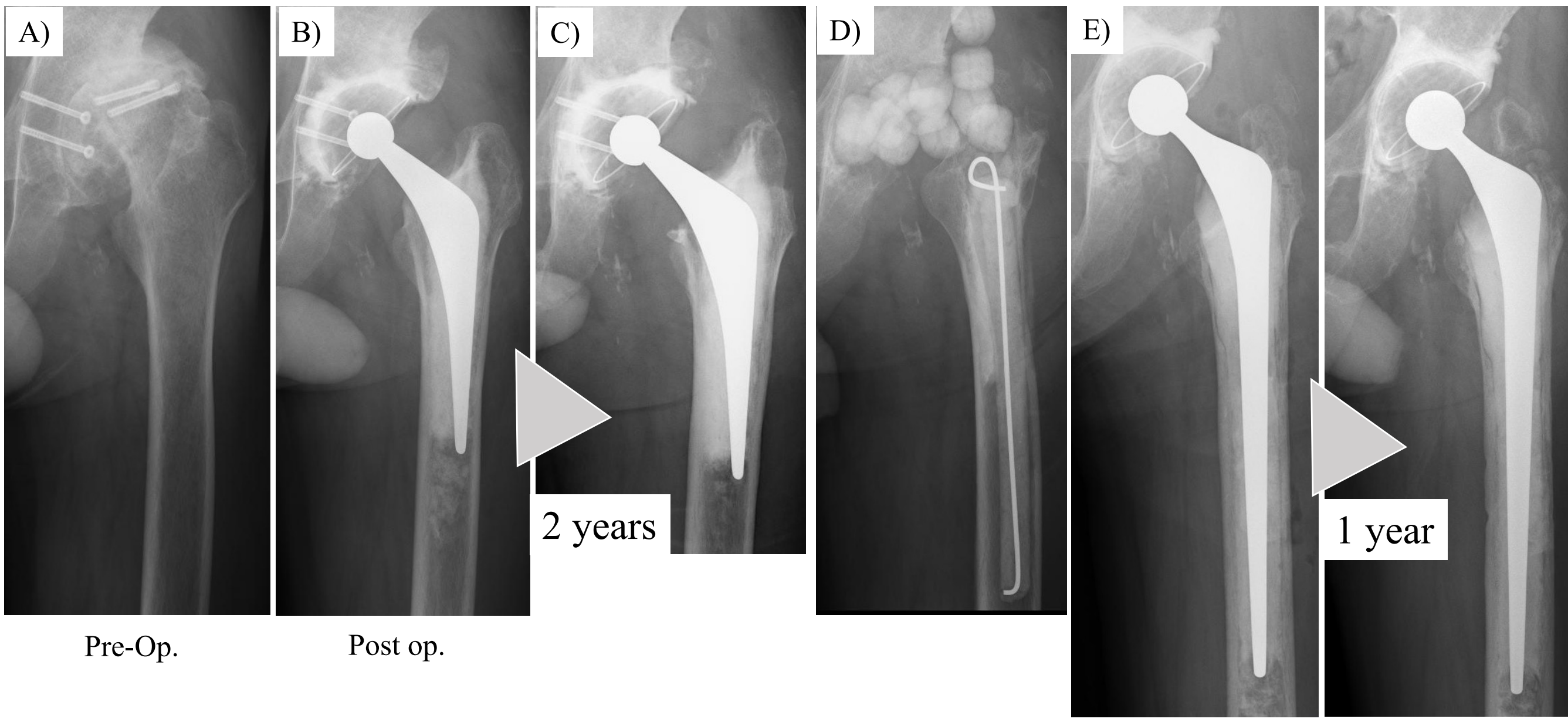

Supplementary Figure S5. Clinical course and radiographic evaluation of Case 7.
A 50-year-old female presented with severe post-traumatic osteoarthritis 15 years after undergoing open reduction and internal fixation (ORIF) for an acetabular fracture, and subsequently underwent primary cementless total hip arthroplasty (THA). At the primary surgery, both histopathology and conventional intraoperative cultures were negative; however, a sample derived from the retained ORIF screws yielded a positive qPCR result (Sample 13; Cq = 24.3). Baseline next-generation sequencing (NGS) of this sample identified *Streptococcus*, *Cutibacterium*, and *Staphylococcus*. Crucially, two years after the primary THA, the patient developed a delayed periprosthetic joint infection (PJI), with cultures confirming *Staphylococcus* as the causative pathogen.
A) Preoperative radiograph showing post-traumatic osteoarthritis with retained internal fixation hardware from the prior ORIF surgery. **B)** Postoperative radiograph following the primary cementless THA. **C)** Radiograph obtained at the onset of delayed PJI, two years after the primary surgery, demonstrating implant loosening. **D)** Radiograph during the first stage of the two-stage revision, showing implant removal and insertion of antibiotic-loaded acrylic cement beads/spacer to eradicate the infection. **E)** Postoperative radiographs obtained immediately after re-implantation (left) and 1 year postoperatively (right), demonstrating stable cemented revision components and successful resolution of the PJI.

Supplementary Table S1.

Next-generation sequencing (NGS) analysis results of Microbial Mock Community B (mock DNA controls HM-782D and HM-783D).

Taxon	HM-782D (Expected)	HM-782D	Taxon	HM-783D (Expected)	HM-783D
g__Acinetobacter	5%	0.09%	*g__Acinetobacter*	0.22%	0.00%
g__Actinomyces	5%	2.53%	*g__Actinomyces*	0.02%	0.01%
g__Bacillus	5%	6.79%	*g__Bacillus*	2.19%	2.77%
g__Bacteroides	5%	8.72%	*g__Bacteroides*	0.02%	0.02%
g__Clostridium	5%	9.30%	*g__Clostridium*	2.19%	2.55%
g__Deinococcus	5%	2.15%	*g__Deinococcus*	0.02%	0.02%
f__Enterobacteriaceae	5%	0.08%	*f__Enterobacteriaceae*	0.02%	0.35%
g__Enterococcus	5%	0.16%	*g__Enterococcus*	21.91%	0.00%
g__Helicobacter	5%	9.59%	*g__Helicobacter*	0.22%	0.21%
g__Lactobacillus	5%	4.27%	*g__Lactobacillus*	0.22%	0.11%
g__Listeria	5%	6.90%	*g__Listeria*	0.22%	0.21%
g__Neisseria	5%	7.78%	*g__Neisseria*	0.22%	0.19%
g__ Cutibacterium	5%	7.14%	*g__ Cutibacterium*	0.22%	0.24%
f__Pseudomonadaceae	5%	0.01%	*f__Pseudomonadaceae*	2.19%	0.01%
g__Rhodobacter	5%	2.40%	*g__Rhodobacter*	21.91%	15.57%
g__Staphylococcus	10%	7.86%	*g__Staphylococcus*	24.10%	36.97%
g__Streptococcus	15%	16.35%	*g__Streptococcus*	24.12%	38.69%
Others		7.88%	*Others*		2.08%
Total	100%	100%	Total	100%	100%

Supplementary Table S2. Taxonomic profiles of positive controls (serially diluted ***Escherichia coli***) and negative controls ranked by relative abundance (Top10).

**Positive control CFU/ml (qPCR (Cq))**	***E. coli*** 10^7 CFU/ml ***(17.9)***	***E. coli*** 10^6 CFU/ml ***(21.5)***	***E. coli*** 10^5 CFU/ml ***(25.5)***	***E. coli*** 10^4 CFU/ml ***(29.1)***
f__Enterobacteriaceae	99%	85%	35%	4%
g__ Cutibacterium	0%	4%	17%	32%
g__Streptococcus	0%	4%	14%	19%
f__Bradyrhizobiaceae	0%	1%	7%	3%
g__Staphylococcus	0%	0%	6%	2%
f__Sphingomonadaceae	0%	0%	2%	6%
g__Sediminibacterium	0%	1%	1%	4%
g__Corynebacterium	0%	1%	3%	1%
f__Sinobacteraceae	0%	0%	1%	2%
f__Comamonadaceae	0%	0%	0%	3%

**Negative control (qPCR (Cq))**	NF (30.8)	Saline (32.0)	Saline (32.4)
g__Sediminibacterium	14%	27%	24%
***o*__MLE1-12**	3%	15%	18%
g__ Cutibacterium	33%	1%	0%
f__Bradyrhizobiaceae	4%	18%	12%
f__Sphingomonadaceae	4%	16%	13%
g__Streptococcus	24%	0%	0%
f__Sinobacteraceae	3%	11%	8%
f__Bradyrhizobiaceae	0%	1%	6%
o__Myxococcales	0%	2%	5%
g__Sphingomonas	0%	4%	1%

E.coli, *Escherichia coli*; Cq, quantification cycle; NF, nuclease-free water; CFU, colony-forming unit.

Note: Bacterial taxa are represented at the family (f__), genus (g__), or order (o__) level based on the operational taxonomic classifications.

Supplementary Table S3.
Correlation between next-generation sequencing relative abundance and qPCR Cq values across high- and low-biomass groups.

Rank*			**Bacteria**	**Correlation coefficient with qPCR**	**p†**
**All samples**	Cq ≤ 25.4 **(High biomass)**	Cq ≥ 25.5 **(Low biomass)**			
1	1	1	*g__Streptococcus*	-0.042	0.814
2	2	2	*g__ Cutibacterium*	0.161	0.37
3			*f__Enterobacteriaceae*	-0.293	0.146
4	4	3	*g__Staphylococcus*	-0.05	0.795
5	6	8	*g__Sediminibacterium*	0.397	0.03
6	3		*o__Streptophyta*	0.165	0.385
7	5	5	*o*__MLE1-12	0.397	0.04
8	7	4	*f__Sinobacteraceae*	0.506	0.05
9	8	7	*f__Sphingomonadaceae*	0.388	0.05
10	10		*f__Bradyrhizobiaceae*	0.283	0.191
		10	*f__Erythrobacteraceae*	0.102	0.572
	9	9	*c*__SM2F11	-0.062	0.736
		6	*o__Actinomycetales*	0.098	0.618
			Unassigned;Other	0.327	0.063

qPCR, quantitative polymerase chain reaction; Cq, quantification cycle; p, P value.
*Rank indicates the relative abundance hierarchy: rank among all samples, rank within the high-biomass group (Cq ≤ 25.4), and rank within the low-biomass group (Cq ≥ 25.5), respectively. Top 10 bacterial taxa were identified to the genus level or highest resolvable taxonomic level.
†Statistical significance at $P < 0.05$.

Supplementary Table S4

Relative taxonomic abundance of the derivation group after organellar filtering and computational decontamination using the R package *decontam*.

Sample No. (Figure 6)	1	2	3	5	7	9	11	13			
PCR (Cq)	17.7	18.7	21	21.4	21.6	23.4	24.023	24.3	30.8	32	32.4
Case No. (Figure 6)	1	1	2	3	4	5	6	7	NF	Saline	Saline
Bacteria											
Streptococcus	100%	100%	20%	15%	0%	32%	44%	63%	30%	0%	0%
Cutibacterium	0%	0%	17%	29%	100%	36%	27%	22%	53%	18%	3%
Staphylococcus	0%	0%	7%	32%	0%	0%	4%	5%	0%	0%	10%
Candidatus Rhodoluna	0%	0%	5%	0%	0%	0%	0%	0%	0%	0%	0%
Burkholderia	0%	0%	7%	0%	0%	3%	0%	0%	0%	0%	0%
Bacillus	0%	0%	0%	0%	0%	0%	8%	0%	0%	0%	0%
Anaerococcus	0%	0%	14%	0%	0%	0%	0%	0%	0%	0%	0%
Hydrogenophilus	0%	0%	0%	0%	0%	0%	0%	0%	0%	0%	8%
Paracoccus	0%	0%	11%	0%	0%	0%	0%	0%	0%	0%	0%
Cloacibacterium	0%	0%	0%	0%	0%	0%	0%	0%	0%	0%	0%
Others	0%	0%	19%	24%	0%	29%	17%	10%	17%	82%	79%

qPCR, quantitative polymerase chain reaction; Cq, quantification cycle; NF, nuclease-free water.
Note: Bacterial genera listed in the table (e.g., *Streptococcus*, *Propionibacterium*, *Staphylococcus*) are presented in italics in accordance with international microbiological standards.

Supplementary Table S5

Next-generation sequencing microbial profile of Case 2 before and after computational decontamination using the R package ***decontam***.

Case 2 (hip prosthesis loosening)

Relative abundance	before	after
Anaerococcus	11%	14%
Burkholderia	5%	7%
Candidatus Rhodoluna	4%	5%
Paracoccus	8%	11%
Cutibacterium	13%	17%
Sediminibacterium	8%	-
Staphylococcus	5%	7%
Streptococcus	15%	20%
Others	31%	19%

Supplementary Table S6.

Next-generation sequencing microbial profiles of Sample 9 and Sample 10 from Case 5 before and after computational decontamination using the R package ***decontam***.

Case 5, one-stage revision THA, suspected septic loosening

Relative abundance	before		after	
Sample No.	9	10	9	10
PCR (Cq)	23.4	30.8	23.4	30.8
Relative abundance				
Burkholderia	-	-	3%	0%
Cutibacterium	32%	12%	36%	12%
Streptococcus	28%	70%	32%	72%
Others	39%	18%	29%	16%

Supplementary Table S7.
Next-generation sequencing microbial profiles of Samples 13, 14, and 15 from Case 7 before and after computational decontamination using the R package *decontam*.

Case 7, primary THA for traumatic coxarthrosis, initially treated as a non-infected case.

	before			after		
Sample No.	13	14	15	13	14	15
PCR (Cq)	24.3	30.0	30.7	24.3	30.0	30.7
Relative abundance						
Propionibacterium	21%	10%	9%	22%	11%	9%
Staphylococcus	5%	0%	0%	5%	0%	0%
Streptococcus	60%	73%	77%	63%	76%	79%
Others	14%	17%	14%	10%	13%	12%
